# Online sources of health-related traditional knowledge: content for a traditional medicine library

**DOI:** 10.2471/BLT.25.293555

**Published:** 2026-03-16

**Authors:** Carmen VM Abdala, Mariana C Schveitzer, Ricardo Ghelman, Mirelys Puerta-Diaz, M Sharmila A Sousa, Viswajanani J Sattigeri, Geetha K Gopalakrishna Pillai, Maki Kajiwara, Shyama Kuruvilla, João P Souza

**Affiliations:** a Latin American and Caribbean Center on Health Sciences Information, Pan American Health Organization, Rua Vergueiro 1759, 12° andar, Paraíso, São Paulo-SP, 04101-000, Brazil.; b Paulista School of Medicine, Federal University of São Paulo, São Paulo, Brazil.; c Department of Medicine on Primary Health Care, Federal University of Rio de Janeiro, Rio de Janeiro, Brazil.; d São Paulo, Brazil; e Council of Scientific & Industrial Research, Traditional Knowledge Digital Library Unit, Ministry of Science and Technology, New Delhi, India.; ^f^ Global Traditional Medicine Centre, World Health Organization, Jamnagar, India.

## Abstract

**Objective:**

To map digital resources on traditional, complementary and integrative medicine, including databases, repositories, libraries and web portals providing access to traditional knowledge, research or policy information.

**Methods:**

We undertook a rapid review of publications related to digital resources on traditional medicine. We also surveyed specialists in traditional medicine for referrals to digital resources. We searched PubMed®, Embase, the Virtual Health Library of the Pan American Health Organization and Google. Eligible resources were digital platforms indexing traditional medicine knowledge, research or policy. From the publications identified, we retrieved relevant digital resources and extracted data on their scope, content and geographic distribution.

**Findings:**

From 102 studies, we identified 358 potentially relevant digital resources on traditional medicine across all regions of the World Health Organization (WHO). We included 125 of these resources in our inventory of traditional medicine digital resources. The Western Pacific Region accounted for 36% (45/125) of the resources, led by China with 34 resources, and the Americas accounted for 24% (30/125) of the resources, with 24 resources from the United States of America. Most digital resources focused on pharmacological or clinical applications; only five addressed Indigenous medicine.

**Conclusion:**

Digital resources on traditional, complementary and integrative medicine are diverse but fragmented. Codified systems are predominant while Indigenous traditions are marginalized. WHO’s Traditional Medicine Global Library offers an opportunity to correct these imbalances by creating an inclusive, ethically governed platform that safeguards knowledge systems and supports their equitable integration into global health.

## Résumé

**Objectif:**

Identifier les ressources numériques sur la médecine traditionnelle, complémentaire et intégrative, y compris les bases de données, référentiels, bibliothèques et portails Web donnant accès à des connaissances traditionnelles, à des recherches ou à des informations en matière de politiques.

**Méthodes:**

Nous avons procédé à un examen rapide des publications liées aux ressources numériques sur la médecine traditionnelle. Nous avons également interrogé des spécialistes en médecine traditionnelle afin d’obtenir des références vers des ressources numériques. Nous avons effectué des recherches dans PubMed®, Embase, la bibliothèque virtuelle de santé de l’Organisation panaméricaine de la Santé et Google. Les ressources éligibles étaient des plateformes numériques répertoriant les connaissances, les recherches ou les politiques en matière de médecine traditionnelle. Les publications identifiées nous ont permis de récupérer les ressources numériques pertinentes et d’extraire des données sur leur portée, leurs contenus et leur répartition géographique.

**Résultats:**

Dans 102 études, nous avons identifié 358 ressources numériques potentiellement pertinentes sur la médecine traditionnelle dans toutes les régions de l’Organisation mondiale de la Santé (OMS). Nous avons inclus 125 de ces ressources dans notre inventaire des ressources numériques sur la médecine traditionnelle. La région du Pacifique occidental représentait 36% (45/125) des ressources, la Chine arrivant en tête avec 34 ressources, et les Amériques représentaient 24% (30/125) des ressources, avec 24 ressources provenant des États-Unis d’Amérique. La plupart des ressources numériques étaient axées sur les applications pharmacologiques ou cliniques. Seules cinq d’entre elles traitaient de la médecine autochtone.

**Conclusion:**

Les ressources numériques sur la médecine traditionnelle, complémentaire et intégrative sont diverses, mais fragmentées. Les systèmes codifiés prédominent, tandis que les traditions autochtones sont marginalisées. La Bibliothèque mondiale de l’OMS sur la médecine traditionnelle offre la possibilité de corriger ces déséquilibres en créant une plateforme inclusive et régie par des principes éthiques qui préserve les systèmes de connaissances et soutient leur intégration équitable dans le système mondial de la santé.

## Resumen

**Objetivo:**

Cartografiar los recursos digitales sobre medicina tradicional, complementaria e integrativa, incluidas bases de datos, repositorios, bibliotecas y portales web que proporcionan acceso a conocimientos tradicionales, investigaciones o información normativa.

**Métodos:**

Se realizó una revisión rápida de publicaciones relacionadas con recursos digitales sobre medicina tradicional. Asimismo, se consultó a especialistas en medicina tradicional para identificar recursos digitales pertinentes. Se realizaron búsquedas en PubMed®, Embase, la Biblioteca Virtual en Salud de la Organización Panamericana de la Salud y Google. Se consideraron elegibles las plataformas digitales que indexaban conocimientos, investigaciones o políticas sobre medicina tradicional. A partir de las publicaciones identificadas, se recuperaron recursos digitales pertinentes y se extrajeron datos sobre su alcance, contenido y distribución geográfica.

**Resultados:**

A partir de 102 estudios, se identificaron 358 recursos digitales potencialmente pertinentes sobre medicina tradicional en todas las regiones de la Organización Mundial de la Salud (OMS). De estos, se incluyeron 125 recursos en el inventario de recursos digitales sobre medicina tradicional. La Región del Pacífico Occidental representó el 36% (45/125) de los recursos, encabezada por China con 34 recursos, mientras que la Región de las Américas representó el 24% (30/125), con 24 recursos procedentes de los Estados Unidos de América. La mayoría de los recursos digitales se centraban en aplicaciones farmacológicas o clínicas; solo cinco abordaban la medicina indígena.

**Conclusión:**

Los recursos digitales sobre medicina tradicional, complementaria e integrativa son diversos, pero fragmentados. Los sistemas codificados predominan, mientras que las tradiciones indígenas permanecen marginadas. La Biblioteca Mundial de Medicina Tradicional de la OMS ofrece una oportunidad para corregir estos desequilibrios mediante la creación de una plataforma inclusiva y regida por principios éticos que salvaguarde los sistemas de conocimiento y apoye su integración equitativa en la salud mundial.

## ملخص

**الغرض:**

تحديد الموارد الرقمية المتعلقة بالطب التقليدي والتكميلي والتكاملي، بما في ذلك قواعد البيانات، والمستودعات، والمكتبات، والبوابات الإلكترونية التي تتيح الوصول إلى المعرفة التقليدية، أو الأبحاث، أو المعلومات المتعلقة بالسياسات.

**الطريقة:**

قمنا بإجراء مراجعة سريعة للمنشورات المتعلقة بالموارد الرقمية حول الطب التقليدي. كما قمنا باستطلاع آراء المتخصصين في الطب التقليدي للحصول على إحالات إلى الموارد الرقمية. قمنا بالبحث في قواعد بيانات ®PubMed وEmbase، والمكتبة الصحية الافتراضية لمنظمة الصحة للبلدان الأمريكية، وGoogle. كانت الموارد المؤهلة عبارة عن منصات رقمية تقوم بفهرسة المعرفة أو الأبحاث أو السياسات المتعلقة بالطب التقليدي. من المنشورات التي تم تحديدها، قمنا باسترجاع الموارد الرقمية ذات الصلة، واستخرجنا بيانات عن نطاقها ومحتواها وتوزيعها الجغرافي.

**النتائج:**

من بين 102 دراسة، قمنا بتحديد 358 موردًا رقميًا محتملًا ذي صلة بالطب التقليدي عبر جميع مناطق منظمة الصحة العالمية (WHO). أدرجنا 125 من هذه الموارد في قائمتنا للموارد الرقمية للطب التقليدي. استحوذت منطقة غرب المحيط الهادئ على %36 (45/125) من الموارد، بقيادة الصين في ظل وجود 34 موردًا، واستحوذت الأمريكتان على %24 (30/125) من الموارد، مع 24 موردًا من الولايات المتحدة الأمريكية. قامت معظم الموارد الرقمية بالتركيز على التطبيقات الدوائية أو الإكلينيكية؛ ولم تتناول سوى خمسة منها الطب التقليدي.

**الاستنتاج:**

الموارد الرقمية المتعلقة بالطب التقليدي والتكميلي والتكاملي متنوعة ولكنها مجزأة. وتسود الأنظمة المقننة بينما يتم تهميش التقاليد التقليدية. توفر المكتبة العالمية للطب التقليدي التابعة لمنظمة الصحة العالمية فرصة لتصحيح هذه الاختلالات، وذلك من خلال إنشاء منصة شاملة تخضع لقواعد أخلاقية تحمي أنظمة المعرفة، وتدعم دمجها بشكل منصف في الصحة العالمية.

## 摘要

**目的:**

旨在了解传统、补充和整合医学相关数字资源,包括提供传统知识、研究或政策信息访问权限的数据库、信息库、图书馆和门户网站。

**方法:**

我们对涉及传统医学数字资源的出版物进行了速览综述。我们还面向传统医学领域的专家开展了调研活动,以了解其推荐使用的数字资源。我们搜索了 PubMed^®^、Embase、泛美卫生组织的虚拟健康图书馆和谷歌 (Google),然后将可检索到传统医学知识、研究或政策的数字平台纳入了适用数字资源的范畴。我们从已确定的出版物中检索到了相关数字资源并提取了有关此类资源范围、内容及地理分布情况的数据。

**结果:**

基于 102 项研究,我们在世卫组织 (WHO) 所有区域内共找到了 358 个可能有关的传统医学数字资源。我们将其中 125 个资源列入了我们的传统医学数字资源清单中。在列入清单的这些资源中,西太平洋区域占 36% (45/125),其中又以中国(34 个资源)的占比最高;美洲区域占 24% (30/125),其中 24 个资源来自于美利坚合众国。大多数数字资源侧重于药理作用或临床应用;仅五个资源涉及土著医学**。**

**结论:**

尽管传统、补充和整合医学相关数字资源种类繁多,但此类资源过于零散,未形成体系。编码化系统逐步占据主导地位,而土著传统则有向边缘化发展的趋势。世卫组织的传统医学全球图书馆创建了一个具有包容性且符合伦理规范的平台来保护知识系统和支持以公平合理的方式将此类知识系统整合到全球健康系统中,从而确保有机会纠正此类失衡现象。

## Резюме

**Цель:**

Составить карту цифровых ресурсов по традиционной, комплементарной и интегративной медицине, в том числе баз данных, репозиториев, библиотек и веб-порталов, предоставляющих доступ к информации о традиционных знаниях, научных исследованиях или политике в данной области.

**Методы:**

Авторы выполнили краткий обзор публикаций, посвященных цифровым ресурсам по традиционной медицине. Были также опрошены специалисты по традиционной медицине, которые могли бы дать рекомендации по цифровым ресурсам. Поиск проводился по базам данных PubMed®, Embase, Виртуальной библиотеке здоровья Панамериканской организации здравоохранения, а также в Google. В рассмотрение включались цифровые платформы, индексирующие информацию по знаниям, соответствующим научным исследованиям и политике в области традиционной медицины. Из выявленных публикаций были извлечены соответствующие цифровые ресурсы и данные об их тематическом охвате, содержимом и географическом распределении.

**Результаты:**

По данным 102 исследований было выявлено 358 потенциально релевантных цифровых ресурсов по традиционной медицине во всех регионах Всемирной организации здравоохранения (ВОЗ). 125 из них авторы включили в перечень цифровых ресурсов по традиционной медицине. На долю Западного Тихоокеанского региона пришлось 36% (45/125) ресурсов, во главе списка идет Китай с 34 ресурсами из них, а на долю Американского континента – 24% (30/125), где 24 ресурса представляли Соединенные Штаты Америки. Большинство цифровых ресурсов были посвящены вопросам фармакологии или клинического применения, и только пять освещали вопросы медицины коренных народов.

**Вывод:**

Цифровые ресурсы по традиционной, комплементарной и интегративной медицине отличаются разнообразием, но в то же время и фрагментированностью. Преобладают кодифицированные системы, тогда как традиции коренных народов остаются на периферии. Глобальная библиотека ВОЗ по традиционной медицине предлагает возможность исправить этот дисбаланс, создав инклюзивную, управляемую на основе этических принципов платформу, которая защищает системы знаний и поддерживает их равноправное включение в систему мирового здравоохранения.

## Introduction

In 2015, government leaders committed to fostering prosperity and sustainability by adopting the sustainable development goals.^1^ Health is a cornerstone of sustainable development, with universal access to good-quality health services essential to achieving the highest standard of health for all. The universal health coverage (UHC) service coverage index, which is a score from 0 to 100 that tracks progress towards UHC, increased significantly from 44.6 in 2000 to 68 in 2021.^2^ However, many countries continue to face challenges in bridging the coverage gap to offer consistent and equitable high-quality services.^3^

In this context, a considerable portion of the global population depends on traditional health practices, either as their main form of health care or as a complement to conventional biomedicine. The World Health Organization (WHO) uses the term traditional medicine as an umbrella concept encompassing traditional, complementary and integrative medicine, and we have used this term similarly in our review. Among the 179 WHO Member States providing information for the *WHO global report on traditional and complementary medicine 2019*, 170 have reported the use of some form of traditional medicine among their population.^4^ Thus, identifying safe, effective and culturally appropriate traditional medicine practices could play an important role in advancing health for all. Furthermore, biodiversity and traditional knowledge are important to pharmaceutical development, with about 40% of existing drugs derived from natural sources. Scientific discovery and bioprospecting informed by traditional knowledge have led to the development of new treatments, such as quinine, artemisinin and many others, highlighting the potential of this combined approach.^5^

With the progressive digital transformation of our societies, digital resources of traditional knowledge and scientific evidence related to traditional health practices become essential for equitable access to this knowledge and to maximize their contribution to global health and well-being. The Virtual Health Library on Traditional, Complementary and Integrative Medicine,^6^ a digital library for scientific and technical information focusing on the WHO Americas Region, and the Traditional Knowledge Digital Library,^7^ a digital repository of Indian traditional knowledge, are well-known examples of such digital resources. These databases serve as valuable repositories of traditional knowledge and related scientific information as well as tools for safeguarding the rights of Indigenous communities and nations. In particular, the Traditional Knowledge Digital Library is safeguarding knowledge by establishing prior art (publicly available evidence before a patent filing date) to prevent misappropriation and biopiracy. In response to requests from Member States, WHO is developing a comprehensive database and knowledge platform that brings together scientific evidence and documented traditional medical knowledge, that is, the WHO Traditional Medicine Global Library.^8^

This library addresses the need for a unified, inclusive platform to overcome the fragmentation, regional disparities and narrow range of topics that currently characterize digital resources on traditional medicine. While many existing databases focus mainly on pharmacological knowledge or codified systems, the Traditional Medicine Global Library aims to consolidate different forms of documented knowledge (scientific, technical, experiential and Indigenous) into a single, accessible resource. The library is designed to serve policy-makers, researchers, health workers, practitioners, educators and community stakeholders, and support regulation, evidence-informed practice, education and ethical use of traditional knowledge. Furthermore, the library seeks to enhance global knowledge equity by promoting the visibility of underrepresented regions and knowledge traditions in the health sciences.

We present the findings of a rapid review of existing digital resources related to traditional medicine and provide an inventory of relevant platforms. For the purposes of this study, traditional medicine digital resources are defined as online databases, digital knowledge repositories, digital libraries, searchable web portals and other internet-based platforms that provide access to knowledge, scientific literature and technical or policy-related information on traditional medicine. The findings of this review, integrated with an incremental and participatory development process, will support the continuous development and implementation of the Traditional Medicine Global Library. The review aims not only to map the current landscape of digital resources on traditional medicine but also to contribute to the ethical, technical and conceptual foundations of this library.

## Methods

We used rapid review methods^9^ and a survey of specialists in traditional medicine to help identify existing digital resources in this field.

### Rapid literature review

We developed an adaptation of a rapid review of the literature to identify traditional medicine digital resources, as this type of evidence synthesis provides timely information to decision-makers (e.g. health-care planners, health workers, policy-makers and patients).^10^^,^^11^ The protocol was registered on the Open Science Framework.^12^ The review began in February 2024 and ended by October 2024.

The guiding question was, “What are the digital databases, repositories, platforms and libraries on traditional, complementary and integrative medicine across the world?”

#### Literature search

We searched PubMed®, the Pan-American Health Organization (PAHO) Virtual Health Library and Embase for articles on digital resources related to traditional medicine (that is, online databases, digital knowledge repositories, digital libraries, searchable web portals and other internet-based platforms that provide access to knowledge, scientific research, and technical and policy information relevant to traditional medicine). We also made a manual search on Google (Google LLC, Mountain View, United States of America) to identify additional traditional medicine resources and other related specialized databases or repositories. We did not apply any language restrictions, and the search strategy is outlined in the protocol and included in the online repository.^13^ The search terms included combinations such as: database OR databases OR “base de datos” OR “base de dados”; repository OR repositories OR repositorio OR repositories; “information system” OR “sistemas de informacion” OR “sistemas de informacao”; “digital library” OR “virtual library” OR “biblioteca digital”; and “digital catalog” OR “online catalog” OR “electronic catalog.” We used automated language tools, complemented by manual checks to ensure accuracy, to translate studies not in English, Spanish or Portuguese. We imported all studies identified into the reference manager software Rayyan^14^ to remove duplicates and select studies between 26 February 2024 and 10 March 2024.

#### Study selection and data extraction

We included studies focused on digital resources that index resource collections relevant to traditional medicine, including their application to health, well-being, policy and practice. We excluded nonrelevant resources, including resources outside health and websites serving only as portals without a structured database or search function. Four authors independently undertook study selection in parallel using Rayyan.^14^ We resolved disagreements by consensus in pairs. We did not use automation tools for screening and selection, and manually excluded all studies.

During full-text evaluation, three authors independently accessed each digital resource mentioned in the retrieved studies and extracted information using a standardized form that captured: resource name; website; description; type of database; type of indexed data; type of traditional medicine; specific area of traditional medicine; WHO region; country of origin; access model; language; and reference(s) where resources were identified. Based on the reviewers’ evaluations, we included only accessible databases. A full list of included and excluded studies is available in the online repository.^13^

Three authors qualitatively analysed and aggregated the extracted data,^15^ which another author then revised.

### Database referral survey

To identify additional digital resources for traditional medicine as part of Traditional Medicine Global Library co-production, we also developed and implemented an online survey (online repository),^13^ requesting specialists in traditional medicine to submit any resources being used globally in the field. We added studies provided by specialists until April 2025.

## Results

**Fig. 1:**
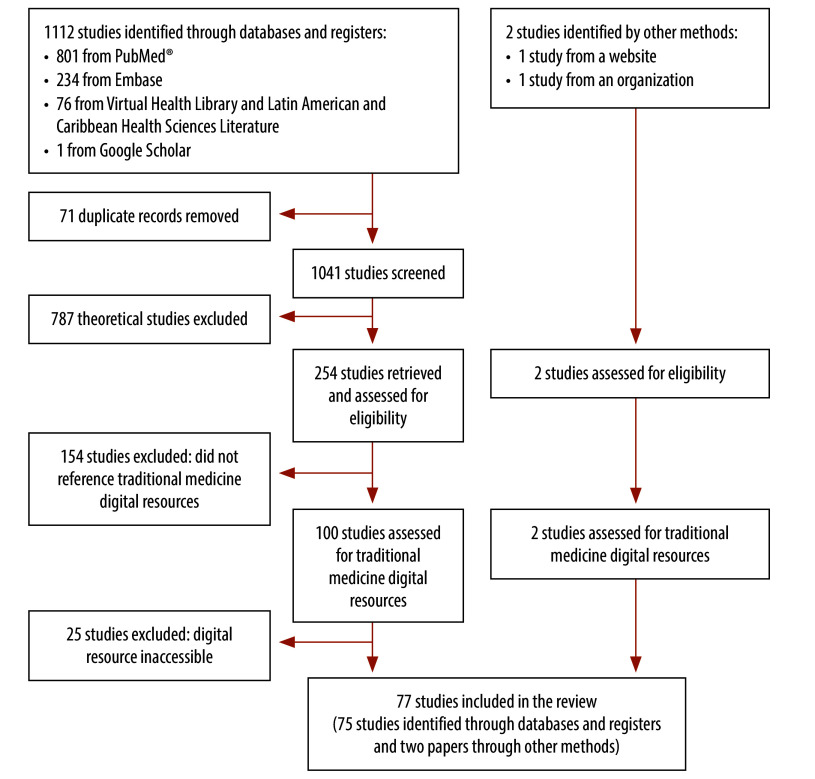
Flowchart of the selection of studies for identifying digital resources on traditional, complementary and integrative medicine

**Fig. 2:**
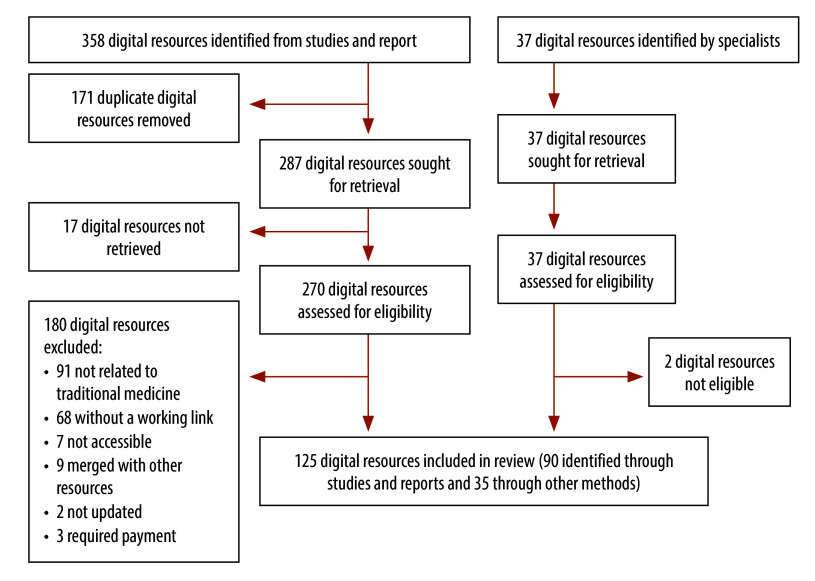
Flowchart of the selection of digital resources on traditional, complementary and integrative medicine

The search retrieved 1112 results. After excluding 71 duplicates, we formally screened 1041 records for title and abstract against eligibility criteria. We retrieved and assessed the full text of 254 articles and excluded 179 studies. Other methods led to the identification, retrieval and assessment of two additional studies (Fig. 1). These 77 (75 + 2) studies enabled us to identify 358 potentially relevant digital resources. Information from experts and Google manual search provided another 37 digital resources. After removing duplicates and excluding noneligible digital resources, we included 125 digital resources in the inventory of traditional medicine digital resources (Fig. 2).

We analysed and categorized the included digital resources based on their primary function(s), scope and geographic distribution.^16^ Of the 125 digital resources included, 71 (57%) were pharmacology or phytochemical databases; 35 (28%) were bibliographic databases; nine (7%) were digital libraries; five (4%) were compendia; and five (4%) were digital repositories. Table 1 summarizes the global distribution of the identified digital resources on traditional medicine. The WHO Western Pacific Region accounted for 36% (45/125) of the resources; followed by 24% (30/125) for the Americas Region; 18% (23/125) for the South-East Asia Region; 18% (22/125) for the European Region; and 2% (2/125) each for the Eastern Mediterranean Region and African Region. Only five digital resources were dedicated to Indigenous medicine from regions outside Asia.

**Table 1:** Geographical distribution of digital resources for traditional, complementary and integrative medicine

WHO region	No. of digital resources (%) (*n* = 125)	Countries (no. of resources)
Africa	2 (2)	Ethiopia (1), South Africa (1)
Americas	30 (24)	Brazil (3), Canada (1), countries in Region of the Americas (2),^a^ United States (24)
South-East Asia	23 (18)	Bangladesh (1), India (20), Thailand (2)
Europe	22 (18)	Belgium (1), Germany (13), Norway (1), Romania (1), Russian Federation (1), Spain (2), Switzerland (1), United Kingdom (3)
Eastern Mediterranean	3 (2)	Egypt (1), Iran (Islamic Republic of) (1), Pakistan (1)
Western Pacific	45 (36)	Australia (1), China (34), Japan (3), Malaysia (1), New Zealand (1), Philippines (1), Republic of Korea (2), Singapore (1), Viet Nam (1)

WHO: World Health Organization.

^a^ These databases focus on Latin America and the Caribbean.

Note: percentages do not sum to 100% due to rounding.

**Fig. 3:**
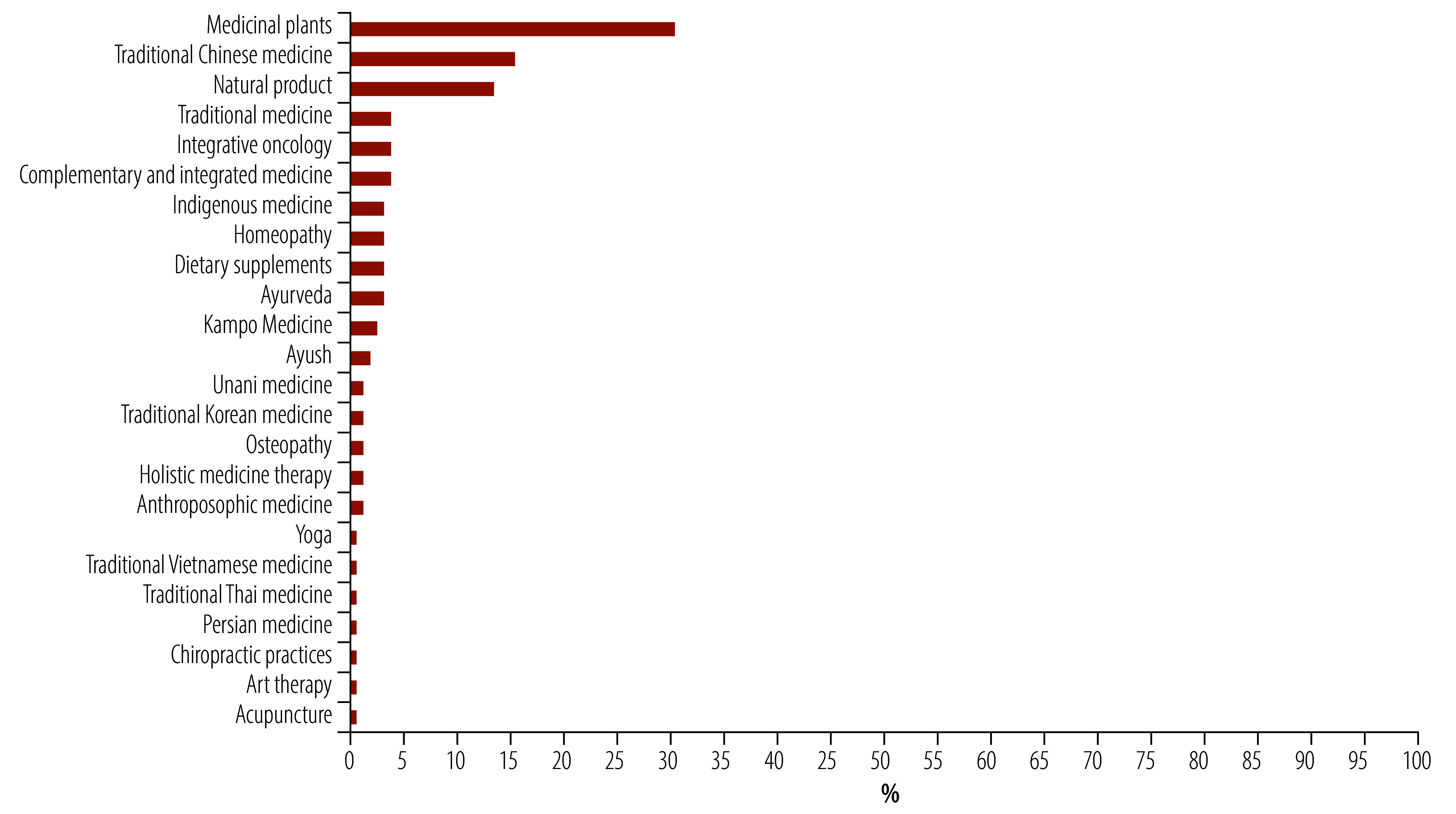
Topic distribution of 125 digital resources on traditional, complementary and integrative medicine

As for countries, 27% (34/125) of traditional medicine digital resources were from China; 19% (24/125) from the United States;16% (20/125) from India; 10% (12/125) from Germany; and 2% (3/125) each from Brazil, Japan and United Kingdom of Great Britain and Northern Ireland. Several countries had two digital resources (Republic of Korea, Spain and Thailand); or one digital resource (Australia, Bangladesh, Belgium, Canada, Egypt, Ethiopia, Islamic Republic of Iran, Malaysia, New Zealand, Norway, Pakistan, Philippines, Romania, Russian Federation, Singapore, South Africa, Switzerland and Viet Nam; Table 2). Fig. 3 shows the topics of traditional medicine covered in the digital resources with some digital resources covering more than one topic. Medicinal plants constitute 38% (47/125), the largest category, followed by 19% (24/125) for traditional Chinese medicine and 17% (21/125) for natural products. Only 5% (6/125) of the digital resources supported general or cross-disciplinary traditional medicine research. A wide range of systems and practices constitute smaller proportions, including integrative oncology, Indigenous medicine, homeopathy, Ayurveda, dietary supplements and various national traditional medicine systems. 

**Table 2:** Distribution of digital resources on traditional, complementary and integrative medicine, by country and topic

Country or region	Digital resources, no.	Traditional medicine	Indigenous medicine	Complementary and integrative medicine therapies
**Country**				
Australia	1	NF	NF	Chiropractic^17^
Bangladesh	1	Medicinal plants^18^	Indigenous knowledge^19^	NF
Belgium	1	Medicinal plants^20^	NF	NF
Brazil	3	NF	Indigenous knowledge^21^	Homeopathy;^22^ natural products^23^
Canada	1	NF	NF	Integrative oncology^24^
China	34	Medicinal plants;^25^^–^^39^ traditional Chinese medicines;^25^^,^^28^^,^^29^^,^^40^^–^^55^ traditional medicine^56^	NF	Integrative oncology;^26^ natural products^34^^,^^36^^,^^57^
Egypt	1	Medicinal plants^58^	NF	NF
Ethiopia	1	Medicinal plants^59^	NF	NF
Germany	12	Traditional Chinese medicines;^60^ Unani medicine;^61^ medicinal plants^62^	NF	Art therapy;^63^ homeopathy;^64^^–^^66^ complementary and integrative medicine;^67^ natural products;^68^^,^^69^ osteopathy;^70^ holistic medicine^71^
India	20	Ayurveda;^7^^,^^72^^–^^75^ Ayush;^7^^,^^76^^,^^77^ medicinal plants;^7^^,^^72^^,^^77^^–^^87^ Unani medicine^7^	NF	Integrative oncology;^88^ natural products^7^^,^^88^^–^^90^
Iran (Islamic Republic of)	1	Iranian traditional medicine^91^	NF	Natural products^91^
Japan	3	Kampo medicine^92^^–^^94^	NF	NF
Malaysia	1	Traditional medicine^95^	NF	Complementary and integrative medicine^95^
New Zealand	1	NF	Indigenous knowledge^96^	NF
Norway	1	NF	NF	Integrative oncology^97^
Pakistan	1	Medicinal plants^98^	NF	NF
Philippines	1	Traditional knowledge^99^	NF	NF
Republic of Korea	2	Medicinal plants;^100^ traditional Korean medicine;^100^^,^^101^ traditional Chinese medicine;^100^ traditional Japanese medicine^100^	NF	NF
Romania	1	Medicinal plants^102^	NF	NF
Russian Federation	1	Medicinal plants^103^	NF	NF
Singapore	1	Traditional Chinese medicine;^104^	NF	NF
South Africa	1	Medicinal plants^105^	NF	Natural products^105^
Spain	2	Medicinal plants^106^	NF	Natural products^107^
Switzerland	1	NF	NF	Anthroposophic medicine^108^
Thailand	2	Medicinal plants;^109^^,^^110^ traditional Thai medicines^110^	NF	NF
United Kingdom	3	NF	NF	Acupuncture^111^; homeopathy;^112^ yoga^113^
United States	24	Medicinal plants;^114^^–^^118^ traditional Chinese medicines;^119^^,^^120^ traditional medicine^121^	Indigenous knowledge ^122^^,^^123^	Anthroposophic medicine^124^; complementary and integrative medicine;^121^^,^^125^^,^^126^ dietary supplements;^114^^,^^117^^,^^118^^,^^126^^–^^130^ holistic medicine and therapies; ^141^ integrative oncology;^114^^,^^131^ natural products;^117^^,^^118^^,^^126^^,^^131^^–^^135^ osteopathic medicine^136^
Viet Nam	1	Medicinal plants;^137^ traditional Vietnamese medicines^137^	NF	NF
**Region**				
Americas^a^	2	Medicinal plants;^138^ traditional medicine^139^	NF	Complementary and integrative medicine^139^

NF: none found.

^a^ Databases focus on Latin American and Caribbean countries.

AYUSH: ayurveda, yoga and naturopathy, Unani, Siddha and homeopathy (plus Sowa-Rigpa).

Note: anthroposophic medicine is a holistic system of medicine combining conventional medicine with spiritual insights; Kampo medicine is traditional Japanese medicine; and Unani medicine is Persian–Arabic traditional medicine. Some digital resources cover more than one topic.

We further categorized the traditional medicine digital resources (Table 2) based on their primary functions. Most resources (53%; 66/125) focused on clinical applications of traditional remedies, including prescriptions, formulations doses, therapeutic uses and drug–herb interactions. An additional 24% (30/125) related to pharmacological research, such as mechanisms of action peptides, targets, formulas and active ingredients for disease treatment; while 20% (25/125) concentrated on component analysis, such as chemical components, structures, compound names, phytochemicals and natural properties. A few sources (3%; 4/125) focused on general research, that is, broad resources supporting general research related to traditional medicine without a specific focus.

The full inventory of the 125 digital resources on traditional medicine identified are presented in Table 3 (available at: https://www.who.int/publications/journals/bulletin). The table gives each platform’s name and bibliographic reference(s) that led to its identification, the traditional medicine area(s) covered, country of origin, brief description, access model (free, log-in restricted or payment required) and primary language(s). We found that the global digital landscape on traditional medicine was heterogenous, ranging from large, well-established pharmacology and medicinal-plant databases and codified systems (for example, traditional Chinese medicine, Ayurveda, Unani, Kampo and Korean medicine) to smaller thematic or population-specific repositories, including a limited number focused on Indigenous knowledge. We also found wide variation in accessibility and linguistic coverage, with many resources openly available and several offering multilingual interfaces, while a smaller number of resources requires subscription or institutional login. Additional information on the digital resources included and the full list of excluded resources with reason for exclusion are available in the online repository.^13^

**Table 3:** Inventory of 125 digital resources on traditional, complementary and integrative medicine, as of 28 February 2025

Name	Topic of traditional medicine	Country or area	Description	Access	Language
About Herbs Database^114^	Medicinal plants; dietary supplements; integrative oncology	United States	A database on common use of herbs and other dietary supplements focused on cancer care, for the public and health-care professionals	Free	English
AcuTrials^®^^119^^,^^140^	Traditional Chinese medicine	United States	A comprehensive collection of randomized controlled trials and systematic reviews of acupuncture published in English	Free	English
Alt HealthWatch^141^	Holistic medicine and therapies	United States	Full-text health research database focused on complementary, holistic and integrated approaches to health care and wellness, offering information on the evolving practice of holistic medicine and therapies	Payment required	English
Allied and Complementary Medicine Database^125^^,^^142^^,^^143^	Complementary and integrative medicine	United States	Designed for physicians, therapists, medical researchers, historians and clinicians looking to learn more about alternative and allied therapies and related subjects	Payment required	English
AMMPDB: Anti Mtb Medicinal Plants Database^72^^,^^144^	Medicinal plants; Ayurveda	India	The database provides traditional and phytochemical data related to Indian medicinal plants that have anti-tuberculosis properties reported in Ayurveda	Free	English
ANPDB: African Natural Products Database^68^^,^^145^	Natural products	Germany	A database of natural products isolated from native organisms in Africa, with data on 6515 compounds from 1042 source organisms, mainly plants, with some microorganisms, animals, and marine sources. The names and molecular structures of the compounds, their biological activities and modes of action are given	Free	English
AnthroMed^124^	Anthroposophic medicine	United States	Repository for articles, audio material and videos relating to Anthroposophic medicine	Free	English
Antimicrobial Peptide Database (APD)^132^^,^^145^	Natural products	United States	A database on antimicrobial peptides with discovery timeline, glossary, nomenclature, classification, structure, function, information search, prediction, design and statistics	Free	English
AromaDb^78^^,^^146^	Medicinal plants	India	Electronic library of aroma molecules of medicinal and aromatic plant origin, with comprehensive information on aroma plants, varieties, accessions, chemotypes, essential oils yields, chromatograms, major and minor compounds, structural elucidation data, structural data of volatile molecules, and biological pathways	Free	English
ARTHEDATA^63^^,^^143^	Art therapy	Germany	A database of literature specific to art therapy, such as monographs, catalogues, essays, abstracts and journal articles	Free	English; German
Arzneipflanzenlexikon^62^^,^^147^	Medicinal plants	Germany	A structured compilation of 180 medicinal plants used in herbal medicines (phytopharmaceuticals) as active ingredients, including information on the medical use of the drugs, such as indications, dosage, side-effects, interactions and warnings	Free	English; German
AntiViral Phyto Chemical Database (AVPCD)^98^^,^^148^	Medicinal plants	Pakistan	Comprehensive resource for viral disease researchers and botanists that aims to bring all antiviral phytochemicals and diseases together from published work especially related to COVID-19, cancer, HIV and malaria	Free	English
Āyurveda Formulation Advanced Database^73^	Ayurveda	India	Ayurveda formulations categorized by their ingredients and methods of preparation used in Ayurveda formulations	Free	English
Ayush Research Portal^76^	Ayush	India	Evidence-based research data of Ayush systems globally	Free	English; Hindi
Bioinformatics Annotation database for Molecular mechanism of Traditional Chinese Medicine (BATMAN-TCM)^25^^,^^145^	Medicinal plants; traditional Chinese medicine	China	Integrative database of known and predicted links between ingredients in traditional Chinese medicine and target proteins. Designed to understand the pharmacological mechanisms of traditional Chinese medicines and identify active ingredients for disease treatment	Free	English
Complementary and Alternative Medicine for Cancer (CAM Cancer)^97^^,^^143^	Integrative oncology	Norway	Summaries of the best available evidence on safety and efficacy of complementary and alternative medicine in cancer care	Free	English
Complementary and alternative medicine database (CAMbase) ^67^^,^^149^^,^^150^	Complementary and integrative medicine	Germany	Literature on complementary and alternative medicine in different resources to support the availability of a systematic overview of relevant literature in the field	Free	English; German
CancerHSP^26^^,^^151^	Medicinal plants; integrative oncology	China	An anticancer herbs database of systems pharmacology which Includes 2439 anticancer herbal medicines with 3575 anticancer ingredients, with the molecular structure and key absorption, distribution, metabolism and excretion (ADME) parameters provided for each ingredient. The anticancer activities of these compounds are based on 492 different cancer cell lines	Free	English; Mandarin
CANNUSE^106^^,^^152^^,^^153^	Medicinal plants	Spain	Provides different aspects of cannabis uses, with more than 2300 data entries from 649 publications related to medicinal, alimentary, fibre and other ethnobotanical cannabis uses from different geographical areas and cultures around the world	Free	English
Computer Access to Research on Dietary Supplements Database (CARDS)^127^^,^^154^	Dietary supplements	United States	Includes federally funded research projects pertaining to dietary supplements	Free	English
ccTCM^40^^,^^155^	Traditional Chinese medicine	China	Provides quantitative analytic data on components and compounds in traditional Chinese medicine, and a variety of online analysis services based on these data	Free	English
Chinese Medicinal Material Images Database^41^^,^^145^	Traditional Chinese medicine	China, Hong Kong SAR	Documents more than 420 crude (unprocessed) drugs commonly used in Chinese medicine practice, providing drug information on source, origin, description, quality, taste and clinical indications, and using high quality photographs to illustrate and identify key properties and selected microstructure of the drugs	Free	English; Mandarin
Chinese Medicine Formulae Images Database^42^^,^^145^	Traditional Chinese medicine	China	Comprises 182 Chinese medicine formulae commonly used in Chinese medical practice, with images and detailed descriptions, including formula composition, processing forms, function, indications and use	Free	English; Mandarin
Chinese Medicine Resource Net^43^^,^^145^	Traditional Chinese medicine	China	Includes old textbooks providing historical insights, traditional prescriptions used in Chinese medicine and acupuncture techniques, as well as information on medicinal teas with a role in herbal therapies	Free; by log-in	Mandarin
Chinese Medicine Specimen Database^44^^,^^145^	Traditional Chinese medicine	China, Hong Kong SAR	A collection of all exhibits of Chinese Materia Medica specimens displayed in the Chinese Medicine Centre	Free	English; Mandarin
Collective Molecular Activities of Useful Plants (CMAUP)^27^^,^^143^^,^^156^	Medicinal plants	China	Provides molecular activities of 7865 plants, including 2954 medicinal plants used in 79 countries or regions, 758 human target proteins and 3013 cancer genetics (gene ontology), 238 KEGG pathways (target proteins of the plant), and their relations to 1399 human diseases	Free	English
Cochrane Complementary Medicine^121^	Traditional medicine; complementary and integrative medicine	United States	Website to support and promote systematic reviews of complementary, traditional, alternative and integrative medicine	Restricted by log-in	English; SP
Clinical Outcome Research in Homeopathy Database (CORE-Hom)^112^^,^^157^	Homeopathy	United Kingdom	Homeopathy database with information on homeopathic research and the quality of studies included	Restricted by log-in	English; German
Database of Unani Medicine^61^	Unani medicine	Germany	Comprises published and unpublished texts, archival material and research literature related to Graeco-Islamic medicine in South Asia, including general research literature on health, medicine and medical ethics in Muslim states and societies in South Asia and other regions	Free	Arabic; English; French; German; Persian; Urdu
Database of Constituents Absorbed into the Blood and Metabolites of Traditional Chinese Medicines (DCABM-TCM)^28^^,^^158^	Medicinal plants; traditional Chinese medicine	China	Includes constituents absorbed into blood and metabolites of traditional Chinese medicine	Free	English
Digital Helpline for Ayurveda Research Articles (DHARA)^74^^,^^159^^–^^162^	Ayurveda	India	Online index of articles on Ayurveda published in research journals worldwide	Free	English
Drug Herb Interaction^45^^,^^163^	Traditional Chinese medicine	China	Promotes quality control of pharmaceutical care and medications and establishes a medication safety environment of Chinese medicine in China	Free	English
Diseases Plants Eliminate (DISPEL)^79^^,^^164^	Medicinal plants	India	Compendium of medicinal plants used for the treatment of infectious as well as noninfectious diseases in humans, with more than 60 000 medicinal plants and associations between medicinal plants and treated diseases including about 5500 medicinal plants and 1000 diseases	Free	English
Dietary Supplements Compendium (DSC)^128^^,^^165^	Dietary supplements	United States	Comprehensive information on ingredient safety and safe limits which supports the development of new dietary supplements, including research, quality control, quality assurance and regulatory and/or compendial matters	Restricted by log-in	English
Dietary Supplement Ingredient Database (DSID)^129^^,^^166^	Dietary supplements	United States	Provides estimated levels of ingredients in dietary supplements sold in the United States	Free	English
Dietary Supplement Label Database (DSLD)^130^^,^^167^	Dietary supplements	United States	Catalogues all information printed on labels of dietary supplement products sold in the United States	Free	English
The National Cancer Institute Natural Products Repository^131^	Natural products; integrative oncology	United States	The world’s largest repository of natural products, housing almost 170 000 extracts from samples of more than 70 000 plants and 10 000 marine organisms collected from more than 25 countries, as well as more than 30 000 extracts of different bacteria and fungi	Free	English
Egyptian herbal monograph^58^	Medicinal plants	Egypt	Includes four databases: Traditional Wild Medicinal Plants; Pharmacopoeial Wild Medicinal Plants; Medicinal Plants used in Egypt; and Herbal Formulations used in Egypt	Free	English
Electroacupuncture. Clinical Studies Database^111^^,^^143^	Acupuncture	United Kingdom	Includes clinical studies on electroacupuncture and other nontraditional acupuncture-based interventions	Free	English
ePlantLIBRA^20^^,^^168^	Medicinal plants	Belgium	A database that comprises information on plant- and plant-food supplements, specifically the bioactive compounds in botanicals and herbal extracts with putative health benefits and adverse effects, giving scientific names, plant families, synonyms, common names in 15 European languages, and colour photographs with details on edible parts, colour, size and shape	Free	English
Encyclopedia of Traditional Chinese Medicine (ETCM)^29^^,^^145^^,^^169^^,^^170^	Medicinal plants; traditional Chinese medicine	China	Comprehensive and standardized information on the commonly used herbs and formulas of traditional Chinese medicine, as well as their predicted target genes, allowing the relationships between traditional Chinese medicine herbs, formulas, ingredients, gene targets and related pathways or diseases to be explored	Free	English
Ethiopian Traditional Medicine Database (ETM-DB)^59^^,^^171^	Medicinal plants	Ethiopia	Includes Ethiopian herbal medicines, related compounds and target genes and/or proteins, as well as phytochemical information	Free	English
1 K Medicinal Plant Genome Database^30^^,^^172^	Medicinal plants	China	Provides sequencing of 1000 important medicinal plant genomes to promote research of genomics, secondary metabolite synthesis biology and related molecular breeding of medicinal plants	Free	English; Mandarin
Global Information Hub on Integrative Medicine (GlobInMed)^95^	Traditional medicine; complementary and integrative medicine	Malaysia	A global electronic resource on traditional and complementary medicine, which includes scientific publications, information about courses and more resources for registered users	Free	English
Global Online Ayurveda Library (GOAL)^75^	Ayurveda	India	A thematic library designed to educate, engage and encourage users to explore the history, philosophy, therapeutic principles, pharmacology and modern advancements in Ayurveda	Free	English
Global Pharmacopoeia Genome Database (GPGD)^31^^,^^145^	Medicinal plants	China	Includes herb genomics data for more than 1000 medicinal species from pharmacopoeias of China, Europe, India, Iran (Islamic Republic of), Japan, Republic of Korea and the USA, with more than 34 000 records from 903 species	Free	English
HERB^46^^,^^170^^,^^173^	Traditional Chinese medicine	China	A large-scale experiment- and reference-guided database of traditional Chinese medicine	Free	English; Mandarin
Herbal Medicine Compendium^115^^,^^165^	Medicinal plants	United States	Contains standards for herbal ingredients used in herbal medicines, primarily in monographs, including general information and the definition of the herbal ingredient related to the monograph title, as well as tests for critical quality attributes of the herbal ingredient, analytical test procedures and acceptance criteria for specified tests	Restricted by log-in	English
Herbal Medicine Database, Thaicrudedrug^109^	Medicinal plants	Thailand	Includes scientific name, chemical composition, properties, and pharmacological, toxicological and clinical studies related to herbal medicines	Free	Thai
Herbal Medicine Omics Database^32^^,^^174^	Medicinal plants	China	Promotes the communication of medicinal plants and related synthetic biology research	Free	English
HerbMed & HerbMedPro^116^^,^^143^	Medicinal plants	United States	Provides hyperlinked access to scientific publications underlying the use of more than 50 of the most popular herbs	Free	English
Herbal Ingredients’ Targets (HIT)^33^^,^^173^^,^^175^^,^^176^	Medicinal plants	China	Comprehensive searching and curation platform for herbal ingredients and target information based on literature evidence	Free	English, Mandarin
Homeopathy Basic Research Experiments database (HomBRex)^64^^,^^177^	Homeopathy	Germany	Three databases covering experimental and clinical studies in homeopathy. Indexes experimental and clinical studies in homeopathy including animal, human, plant, fungi and microbial organisms	Free	German
Homeoindex^22^^,^^178^	Homeopathy	Brazil	Provides scientific and technical information on homeopathy	Free	English; Portuguese; Spanish
Veterinary Clinical Research Database in Homeopathy (HomVetCR)^65^^,^^179^	Homeopathy	Germany	Contains 476 experiments in homeopathic veterinary clinical research	Restricted by log-in	English
Index to Chiropractic Literature^17^^,^^180^	Chiropractic	Australia	Includes peer-reviewed literature produced by chiropractic publishers with links to chiropractic organizations, and links to high-quality online resources in chiropractic	Free	English
Indian Medicinal Plants, Phytochemistry and Therapeutics (IMPPAT)^80^^,^^181^	Medicinal plants	India	Provides information on 1742 Indian medicinal plants, 9596 phytochemicals and 1124 therapeutic uses	Free	English
Integrated Traditional Chinese Medicine (ITCM)^47^^,^^145^	Traditional Chinese medicine	China	Collects and analyses uniform high-throughput sequencing data sets, including 1488 high-quality pharmacological transcription profiles of 496 active ingredients in traditional Chinese medicine	Free	English
International Traditional Medicine Clinical Trial Registry (ITMCTR)^56^^,^^143^	Traditional medicine	China	A nonprofit online resource of clinical trials being conducted in the field of traditional medicine across the world	Free	English
KNOW (Knowledge in Integrative Oncology Website) Database^24^^,^^182^^,^^183^	Integrative oncology	Canada	Provides data on tumour type, natural therapy, conventional treatment and side-effects	Restricted by log-in	English
Lexi-Natural Products^133^^,^^184^	Natural products	United States	A comprehensive, peer-reviewed database providing evidence-based information on more than 450 natural products, herbs and dietary supplements. It is designed for health workers, offering in-depth, structured monographs including dosage, pharmacology, interactions, toxicology and clinical data	Payment required	English
Thai Medicinal Plant Recipe Database (MANOSROI III)^110^^,^^159^^–^^161^^,^^185^^–^^188^	Medicinal plants; Thai traditional medicine	Thailand	Includes Thai medicinal plant recipes from several regions of Thailand, with 723 medicinal textbooks recipes, searchable by recipe, disease, symptoms of the diseases and herbal plants	Free	English; Thai
Medline Plus. Herbs and Supplements^117^	Medicinal plants; natural products; dietary supplements	United States	A repository of herbal medicines and complementary therapies	Free	English; Spanish
Medicinal Fungi Secondary Metabolite And Therapeutics (MeFSAT)^89^^,^^189^	Natural products	India	Includes 184 medicinal fungi with information on their 1830 secondary metabolites and their 149 therapeutic uses	Free	English
Mistletoe-Therapy^108^	Anthroposophic medicine	Switzerland	Provides an overview of scientific studies on the clinical application of mistletoe therapy for cancer, including drug safety, quality of life, combination with other therapies and clinical evidence (overall survival, tumour response)	Free	English; German; Spanish
Medicinal Materials DNA Barcode Database (MMDBD)^34^^,^^145^	Medicinal plants; natural products	China	Provides DNA sequences of medicinal materials and information on and key references for the medicinal materials in the Pharmacopoeia of China, American Herbal Pharmacopoeia and other references	Free	English
MOSAIC^139^	Traditional medicine; complementary and integrative medicine	Countries in the Region of the Americas^a^	Includes conventional and nonconventional documents on traditional, complementary and integrative medicine and related practices in the Region of the Americas	Free	English; Portuguese; Spanish
MPDB 2.0^18^^,^^19^^,^^148^^,^^190^	Medicinal plants; Indigenous knowledge	Bangladesh	Provides phytochemical data on more than 500 indigenous medicinal plants in Bangladesh, giving the plant’s scientific name, family name, local names, parts used and active compounds	Free	English
Myrica rubra database^35^^,^^191^	Medicinal plants	China	Database on Chinese bayberry (*Myrica rubra* Sieb. & Zucc.), extracts of which contain antioxidants against inflammation, allergies, diabetes, cancer, bacterial infections and diarrhoea, among other health issues	Free	English
Natural Product–Drug Interaction Research Database (NaPDI)^134^^,^^145^^,^^192^	Natural products	United States	Examines natural product–drug interactions to determine the clinical relevance of pharmacokinetic interactions between natural products and conventional medications	Free	English
Natural Products ^13^C NMR Database (NAPROC-13)^107^^,^^145^	Natural products	Spain	Provides different search tools to determine the structure of compounds of natural products	By log-in	English; French; German; Italian; Portuguese; Spanish
Native American Ethnobotany^122^^,^^143^	Indigenous knowledge	United States	Documents plants used as drugs, foods, dyes, fibres and more by Indigenous Peoples of North America	Free	English
Native Health Database^123^	Indigenous knowledge	United States	Includes bibliographic information and abstracts of health-related articles, reports, surveys and other resource documents related to health and health care of Indigenous Peoples of North America for the benefit, use and education of organizations and individuals with an interest in health-related issues, programmes and initiatives for these peoples	Free	English
NatMed Pro (previously Natural Standard)^126^^,^^143^	Complementary and integrative medicine; dietary supplements; natural products	United States	Provides information on dietary supplements, natural medicines, and complementary, alternative and integrative therapies based on objective and unbiased evidence	Payment required	English
North East India medicinal plants database (NEI-MPDB)^81^^,^^193^	Medicinal plants	India	Provides an extensive list of medicinal plants used in the north-eastern part of India, including medicinal plants specific to each state, the parts used, method of use, ailments treated and phytochemical constituents of the plants	Free	English
Natural Products Magnetic Resonance Database (NP-MRD)^135^^,^^145^	Natural products	United States	Contains nuclear magnetic resonance spectra and structural data for all known natural products, such as vitamins, minerals, and probiotics as well as small molecules derived from plants, fungi, bacteria, marine organisms and animals	Free	English
Naturally occurring plant-based anticancerous compound-activity-target database (NPACT)^88^^,^^145^	Integrative oncology; natural products	India	Includes natural compounds found in plants that exhibit anticancer activity and has 1574 entries, with each record providing information on the compound’s structure and properties (physical, elemental and topological), cancer type and cell lines against which it has activity, inhibitory values, molecular targets, commercial suppliers and drug-like properties of compounds	Free	English
Natural Product Activity and Species Source Database (NPASS)^36^^,^^145^	Medicinal plants; natural products	China	Includes species source of natural products and connects natural products to biological targets via experimental quantitative activity data; includes 94 413 unique natural products isolated from 32 287 source organisms with 958 866 activity records on 7753 targets	Free	English
New Zealand’s Māori Centre of Research Excellence (NPM)^96^	Indigenous knowledge	New Zealand	Provides research of relevance to Māori communities, including research reports since 2002, videos, short documentaries, social media clips, seminars, keynotes and presentations, books, MAI Journal, AlterNative, E-PANUI (monthly newsletter), and AROTAHI papers	Free	English; Māori
NuBBE^23^^,^^145^	Natural products	Brazil	A database that contains a variety of natural products isolated from Brazilian biodiversity and provides information on chemical (metabolic class, chemical structure, physicochemical properties), biological (species, geographic location, biological activities), pharmacological and spectroscopic data.	Free	English; Portuguese
OSTLIB^70^^,^^194^	Osteopathy	Germany	Includes the entire spectrum of osteopathic studies and professional articles	Free	English
Osteopathic Medical Digital Repository (OSTMED.DR)^17^^,^^136^^,^^195^	Osteopathic medicine	United States	An osteopathic medicine digital library that builds on OSTMED®, a bibliographic index to the literature of osteopathic medicine	Free	English; French; German; Korean; Mandarin; Spanish
PharmDB-K^101^^,^^196^	Traditional Korean medicine	Republic of Korea	Provides comprehensive information on drugs (compounds) related to traditional Korean medicine, disease indication and protein relationship	Free	English; Korean
PlantMolecularTasteDB^102^^,^^145^	Medicinal plants	Romania	Integrates data on orosensorially active phytochemicals (sweet, sour, bitter, salty, umami, pungent, astringent), their chemical and sensorial profile, and their biological activities, such as anti-inflammatory activity	Free	English
PlantPepDB^82^^,^^145^	Medicinal plants	India	Includes plant peptides with different functions and therapeutic activities, with 3848 peptide entries collected from 11 databases and 835 published research articles. Information provided includes peptide sequence, functional information, source, physicochemical properties and tertiary structure	Free	English
ProBioQuest^57^^,^^197^	Natural products	China	Provides up-to-date literature related to probiotics	Free	English
Prediction Tool for Plant-derived Antimicrobial Peptides (PTPAMP)^83^^,^^145^	Medicinal plants	India	Allows prediction of the antimicrobial activity of plant-derived peptides and categorizes results as antimicrobial, antibacterial, antifungal and antiviral activities	Free	English
Qigong and Energy Medicine Database™^120^^,^^143^^,^^198^	Traditional Chinese medicine	United States	Contains around 20 000 abstracts of documents collected by the Qigong Institute since 1984	Free	English
Repositório Saúde dos Povos Indígenas^21^	Indigenous knowledge	Brazil	Includes publications, multimedia and news on the health of Indigenous Peoples in Brazil	Free	Portuguese
South African Natural Compounds Database (SANCDB)^105^^,^^145^	Medicinal plants; natural products	South Africa	Comprises compounds isolated from plant and marine life in and around South Africa, the extracts of which have displayed antimicrobial, anticancer and antidiabetic activity, as well as activity against various neurological disorders	Free	English
Societas Medicinae Sinensis Literature Database^60^^,^^143^	Traditional Chinese medicine	Germany	Provides access to more than 3000 specialist articles in the field of Chinese medicine	Free	English; German; Mandarin
SuperNatural 3.0^69^^,^^145^	Natural products	Germany	Documents natural products and natural product-based derivatives and contains 790 096 different structures (including isomers) and 449 058 unique natural compounds with their structural and physicochemical information. Additional information includes pathways, mechanism of action, toxicity, and vendor information, if available	Free	English
Discover Supplement–Drug Interactions (SUPP.Al)^118^^,^^145^	Medicinal plants; natural products	United States	Presents evidence of supplement and drug interactions extracted from the scientific literature	Free	English
Seaweed Metabolite Database (SWMD)^90^^,^^145^	Natural products	India	Includes secondary metabolites from seaweeds with 1191 seaweed compounds, mostly from the red algae *Laurencia* spp., and comprehensive information on geographical origin, extraction and biological activity	Free	English
SymMap^48^^,^^199^	Traditional Chinese medicine	China	Integrates traditional Chinese medicine with modern medicine through internal molecular mechanism and external symptom mapping, thus providing information on herbs and ingredients, targets, clinical symptoms and diseases treated	Free	English
TCM Database@Taiwan^49^^,^^169^^,^^170^^,^^200^^–^^204^	Traditional Chinese medicine	China, Taiwan	Contains three-dimensional structural information on 37 170 traditional Chinese medicine compounds from 352 different herbs, animal products and minerals, including chemical name, traditional Chinese medicine name, molecular properties and molecular structure	Free	English
TCM2COVID^50^^,^^205^^,^^206^	Traditional Chinese medicine	China	Documents more than 280 anti-COVID traditional Chinese medicine formulas and more than 80 natural products related to 300+ herbs, and describes mechanisms of action of new anti-COVID traditional Chinese medicines	Free	English
TCMBank^145^^,^^207^	Traditional Chinese medicine	China	Provides standardized information on traditional Chinese medicines, ingredients, diseases and their corresponding gene targets	Free	English
DNA Barcoding System for Identifying Herbal Medicine^37^^,^^145^	Medicinal plants	China	Provides a species identification module for herbal materials, internal transcribed spacer 2 and psbA–trnH are the core and supplementary DNA barcodes, respectively, selected for herbal materials	Free	English; Mandarin
Traditional Chinese Medicine Information Database (TCM-ID)^104^^,^^170^^,^^208^	Traditional Chinese medicine	Singapore	Facilitates the research and clinical investigations of traditional Chinese medicine, the authentication of herbs used in traditional Chinese medicine, and the link to health-care big data (via ICD-11 traditional medicine condition codes). The database includes 7443 traditional Chinese medicine prescriptions with information on medicine name, composition, functions and symptoms	Free	English
Traditional Chinese Medicine on Immuno-Oncology (TCMIO)^51^^,^^145^^,^^209^	Traditional Chinese medicine	China	Explores the molecular mechanisms of traditional Chinese medicine in modulating the cancer immune microenvironment	Free	English
TCMPG^52^^,^^145^	Traditional Chinese medicine	China	Provides genome data of plants used in traditional Chinese medicine and computational tools for researchers to perform systematic analysis, with 160 kinds of plants, 195 corresponding genomes and 255 herbs with the related information on plant species, genomes and herbal medicines included	Free	English
Traditional Chinese Medicine Database and Analysis Platform (TCMSP)^53^^,^^170^	Medicinal plants; traditional Chinese medicine	China	A pharmacology platform of Chinese herbal medicines that captures the relationships between drugs, targets and diseases. The database includes chemicals, targets and drug-target networks, and associated drug-target-disease networks, as well as pharmacokinetic properties for natural compounds involving oral bioavailability, drug-likeness, intestinal epithelial permeability, blood–brain barrier, aqueous solubility	Free	English; Mandarin
The Phyto4Health^103^^,^^210^	Medicinal plants	Russian Federation	A database of phytocomponents from plants in the Russian Pharmacopoeia that contains 3128 unique structures of chemical compounds, 233 taxonomic species of 71 families of official Russian medicinal plants, 9489 plant part and phyto component data on interactions with 802 human molecular targets and prediction of biological activities	Free	English
TIPdb^38^^,^^145^	Medicinal plants	China, Taiwan	Includes anti-cancer, anti-platelet and anti-tuberculosis phytochemicals from indigenous plants in China, Taiwan with their chemical structures	Free	English
Traditional Knowledge Digital Library (TKDL)^7^^,^^211^	Ayurveda; Ayush; Medicinal plants; natural products; Unani medicine	India	Contains about 1250 formulations selected from various classical texts of Indian systems of medicine, which use ingredients of plant, animal or mineral origin to treat certain disease conditions	Free	English; French; German; Japanese; Spanish
TM-MC 2.0^100^^,^^170^^,^^212^	Medicinal plants; traditional Chinese medicine; traditional Korean medicine; Kampo medicine	Republic of Korea	Includes information on chemical compounds of medicinal materials listed in Korean, Chinese and Japanese pharmacopoeias, with their corresponding identifiers and pharmacokinetic properties, and information on prescriptions, gene targets, diseases and their associations	Free	English
Program of Applied Research to Popular Medicine in the Caribbean (TRAMIL)^138^	Medicinal plants	Countries in the Region of the Americas^b^	Contains photographs, scans, drawings, a herbarium, thin-section specimens and 100 monographs of 360 medicinal Caribbean plants	Free	English; French; Spanish
UNaProd^91^^,^^213^	Persian medicine; natural products	Iran (Islamic Republic of)	Comprises information on natural products used in Iranian traditional medicine, including medicinal uses, adverse effects, preparation, alternatives, dosage, origin, common name and scientific name	Free	Arabic; English
Veterinary Clinical Research Database for Homeopathy (VetCR)^66^^,^^214^	Homeopathy	Germany	Contains 200 entries of randomized clinical trials, nonrandomized clinical trials, observational studies, drug provings, case reports and case series related to homeopathic medicine, with 22 clinical fields covered	Restricted by log-in	English; German
VIETHERB^137^^,^^215^	Medicinal plants; traditional Vietnamese medicine	Viet Nam	Includes information about various herbs used in traditional Vietnamese medicine as well as related information such as metabolism, diseases, morphology and geographical location of each species	Free	Vietnamese
Wanfang data, China Medical Collections^54^^,^^143^^,^^216^	Traditional Chinese medicine	China	Provides Chinese medicine literature and documentation covering medical journals, dissertations, conference proceedings, patents, standards, companies and products	Free	English; Mandarin
Anti Mtb Medicinal Plants Database (AMMPDB)^84^^,^^144^	Medicinal plants	India	Contains Indian medicinal plants with antitubercular properties reported in Ayurveda	Free	English
Arctium lappa Database^39^^,^^217^	Medicinal plants	China	Provides information on *Arctium lappa* (commonly known as burdock), a food and medicinal plant, including data on burdock’s genome, transcriptome, metabolic pathways, phenotype variation and chemical composition	Free	English
Chinese Herbal Dictionary^55^^,^^145^	Traditional Chinese medicine	China	A bilingual dictionary including herbal usage and toxicity/warnings, and Latin (botanical) names and common names of herbs	Free	English; Mandarin
DATADIWAN^71^^,^^198^	Holistic medicine and therapies	Germany	Provides extensive information on holistic medicine and alternative-science	Free	English; German
Yoga Therapy Library^113^^c^	Yoga	United Kingdom	An online resource hub for yoga professionals, mental health professionals and medical professionals	Payment required	English
KampoDB^92^^c^	Kampo medicine	Japan	Provides information about natural compound–protein interactions predicted through docking simulations and machine learning, and what pathways and biological processes are predicted to be affected by Kampo formulas and crude drugs	Free	English; Japanese
ETHMEDmmm^93^	Kampo medicine	Japan	Includes scientific information on crude drugs and the crude drug specimens stored in the Museum of Materia Medica	Free	English; Japanese
TradMPD^94^^c^	Kampo medicine	Japan	Provides scientific information on crude drugs, Kampo formulas and natural compounds, and includes the results of genetic (crude drug only), chemical and biological analyses on crude drugs, Kampo formulas and natural compounds	Free	English; Japanese
Indian Medicinal Plants Nomenclature Database^85^^c^	Medicinal plants	India	Covers the natural resources used by the Indian system of medicine such as botanical and local names, geodistribution data, maps, propagation and trade information, and stores 7637 botanical names with 119 183 local names from 12 languages across India and 2688 plant images	Free	English
Traded Medicinal Plant Database^86^^c^	Medicinal plants	India	Includes 960 medicinal plant species forming the source of 1289 botanical raw drugs in trade in India	Free	English
EIACP Resource Partner on Biodiversity^87^^c^	Medicinal plants	India	Provides information on medicinal plants, including traditional uses	Free	English
Indian Medicinal Plants Database^77^^c^	Medicinal plants, Ayush	India	Includes 7263 botanical names, more than 150 000 local names in 10 Indian languages and more than 5000 images of Indian medicinal plants related to specific botanical entities	Free	English
Philippine Traditional Knowledge Digital Library on Health^99^^c^	Traditional knowledge	Philippines	Includes ethnopharmacological documentation, traditional healing practices (including rituals), plant compendium, directory of traditional healers, traditional healing terminologies from ethnobotanical studies, old lexicographic and linguistic texts, and current research being conducted in selected ethnolinguistic groups	Free	English

COVID-19: coronavirus disease 2019; DNA: deoxyribonucleic acid; HIV: human immunodeficiency virus; ICD-11: International Classification of Diseases 11th revision.

^a^ Database covers Latin American and Caribbean countries.

^b^ Database covers Caribbean countries.

^c^ Resources that were referred to the review team during the manuscript review.

## Discussion

We compiled an inventory of 125 digital resources on traditional medicine through this rapid review. These resources included online databases, digital knowledge repositories, digital libraries, searchable web portals and other internet-based platforms that provide access to traditional knowledge, scientific literature and technical or policy-related information. 

Our results indicate an emphasis on pharmacognosy and compound discovery within traditional medicine digital documentation. Our results also reveal an important gap in the global visibility, accessibility and preservation of diverse health knowledge systems as few resources focused on Indigenous medicine from regions outside Asia. Our findings are consistent with previous studies highlighting the global research emphasis on bioprospecting and the pharmacological study of natural products.^218^

Bibliometric analyses have demonstrated an increase in publications related to traditional medicine over the past three decades, which reflects a broader interest in the evidence-based integration of traditional medicine into modern health systems.^219^^,^^220^ The predominance of digital resources on Chinese and Ayurvedic medicine corresponds with well-established trends in the scientific literature, where codified traditional medical systems are more frequently represented. Nevertheless, national efforts to establish digital repositories, such as repositories in the Philippines and Thailand, demonstrate emerging initiatives to document different medical traditions. Despite such efforts, our findings point to persistent inequities in digital representation, particularly regarding Indigenous medicine from the Americas and African traditional medicine, which remain largely absent from digital knowledge platforms.^221^^,^^222^ This imbalance reflects broader analyses of exclusion and underrepresentation of knowledge systems in the global health knowledge landscape.

The thematic focus of the digital resources identified further illustrates the dominance of pharmacological and botanical approaches. This pattern reflects a research landscape shaped by bioprospecting priorities and the biomedical framing of traditional knowledge, which often favours discrete bioactive components over holistic systems of care. This finding highlights the lack of platforms that integrate different forms of evidence across traditions and methods. The underrepresentation of Indigenous medicine may result from both knowledge-system barriers, such as favouring European and North American validation criteria, and structural challenges, including limited digitization initiatives, language exclusion and the historical marginalization of Indigenous knowledge systems.^221^^–^^223^ These findings underscore the need to broaden the scope of digital documentation on traditional medicine to include more diverse knowledge frameworks and community-led approaches, especially if digital platforms are to support equitable access, policy development and ethical knowledge-sharing.

Beyond information gaps, this review highlights the ethical and legal complexities associated with the digitization and use of traditional health knowledge. Many digital resources do not explicitly state the terms of access, consent or benefit-sharing, thus raising concerns about biopiracy and the misappropriation of Indigenous knowledge. The historical exclusion of Indigenous Peoples and local communities from decision-making processes related to knowledge governance continues to influence who can access traditional knowledge and under what conditions. As WHO advances the continuous development of the Traditional Medicine Global Library, the establishment of safeguards will be essential to ensure free, prior and informed consent, equitable access, appropriate attribution and mechanisms for benefit-sharing, which are aligned with the Nagoya Protocol and the principles outlined in the Gujarat Declaration.^223^ Initiatives such as the Traditional Medicine Global Library vault, which will provide a secure digital infrastructure for the long-term preservation of traditional knowledge, are important steps to protecting intellectual sovereignty. By enabling traceable metadata, controlled access and culturally sensitive governance, such platforms can promote responsible knowledge-sharing and ensure fair recognition and compensation for Indigenous Peoples and local communities.

A key strength of our rapid review is its systematic and transparent approach to identifying digital resources of traditional medicine across multiple regions, knowledge systems and thematic topics. The review provides a timely mapping of the digital landscape, which can inform the development of the WHO Traditional Medicine Global Library. The combination of electronic searches and expert consultation allowed for identification of a broad range of resources, despite the recognized constraints. However, several limitations to the review must be acknowledged. First, the review focused on digital and internet-retrievable resources and excluded nondigitized, locally maintained and oral knowledge repositories, such as community pharmacopoeias, materia medica and cultural archives. These resources may contain valuable information on traditional medicine not captured in formal databases.^223^ Second, we excluded potentially relevant resources due to broken links or restricted access, which limited comprehensiveness and illustrates the precarious nature of digital permanence. Third, the limited added value of expert consultation suggests that significant portions of traditional knowledge remain either inaccessible, nondigitized or structurally excluded from indexed platforms, which underscores broader challenges of visibility and knowledge equity in global knowledge systems.^219^ Finally, the digital resources were accessed until 28 February 2025, when we first submitted our paper for publication, but subsequently some links were no longer accessible by the time of publication. This work should therefore be interpreted as a snapshot of databases available in the first quarter of 2025. The observed loss of database accessibility within a single year underscores the need for sustainable mechanisms to ensure the long-term digital preservation of resources that may inform health systems and health policy. The digital preservation of traditional knowledge presents particular challenges, especially for initiatives that lack stable, long-term funding.

In addition to mapping the global landscape of traditional medicine digital resources, our review offers insights to inform the technical, conceptual and ethical design of the WHO Traditional Medicine Global Library. The classification of resources by thematic topic, content type and geographic origin contributes to the development of an ontology and metadata structure capable of supporting interoperability across different knowledge systems. Importantly, the identification of gaps, such as the underrepresentation of Indigenous knowledge and the dominance of pharmacognosy, highlights the need for inclusive data models that support diverse epistemic evidence and nonbiomedical frameworks. These findings can also guide the formulation of data governance protocols and access layers within the Traditional Medicine Global Library, including provisions for resource attribution, culturally sensitive access restrictions and the secure storage of protected or sensitive knowledge. To address the prevailing bias towards pharmacological data, the Traditional Medicine Global Library must actively manage different forms of evidence, including Indigenous healing practices, nonpharmacological interventions and community-based health models. Expanding access to underrepresented traditional medicine customs, particularly Indigenous and African traditional medicine, will require targeted digitization initiatives and equitable partnerships with knowledge holders. Moreover, the library should support the ethical and evidence-informed integration of traditional medicine into health systems by enabling different research methods, facilitating policy dialogue and safeguarding traditional knowledge through secure tools such as the library’s vault. Collectively, these measures will help ensure that the Traditional Medicine Global Library evolves into a dynamic, inclusive platform that fosters equitable knowledge-sharing, strengthens regulatory and research capacity, and advances global health equity.

## References

[1] Transforming our world : the 2030 Agenda for Sustainable Development, A/RES/70/1 [internet]. New York: United Nations General Assembly; 2015. Available from: https://www.un.org/en/development/desa/population/migration/generalassembly/docs/globalcompact/A_RES_70_1_E.pdf [cited 2025 Feb 3].

[2] The sustainable development goals report 2024. New York: United Nations; 2024. Available from: https://unstats.un.org/sdgs/report/2024/ [cited 2025 Feb 3].

[3] Darrudi A, Ketabchi Khoonsari MH, Tajvar M. Challenges to achieving universal health coverage throughout the world: a systematic review. J Prev Med Public Health. 2022 Mar;55(2):125–33. 10.3961/jpmph.21.542 35391524 PMC8995934

[4] WHO global report on traditional and complementary medicine 2019. Geneva: World Health Organization; 2019. Available from: https://iris.who.int/handle/10665/312342 [cited 2025 Feb 3].

[5] Tu Y. Artemisinin – a gift from traditional Chinese medicine to the world (Nobel Lecture). Angew Chem Int Ed Engl. 2016 Aug 22;55(35):10210–26. 10.1002/anie.201601967 27488942

[6] The virtual health library on traditional, complementary, and integrative medicine (VHL TCIM). São Paulo: Latin American and Caribbean Center on Health Sciences Information; 2018. Available from: https://mtci.bvsalud.org/en/ [cited 2025 Feb 28].

[7] Traditional knowledge digital library [internet]. New Delhi: Traditional Knowledge Digital Library Unit; 2001. Available from: https://tkdl.res.in/tkdl/langdefault/common/Home.asp?GL=Eng [cited 2025 Feb 28].

[8] WHO traditional medicine global library. Geneva: World Health Organization; 2025. Available from: https://www.tmgl.org/ [cited 2025 Feb 28].

[9] Denzin N, Lincoln Y. Handbook of qualitative research. Thousand Oaks: Sage Publication, Inc.; 1994.

[10] Tricco AC, Langlois E, Straus SE, editors. Rapid reviews to strengthen health policy and systems: a practical guide. Geneva; World Health Organization; 2017. Available from: https://iris.who.int/handle/10665/258698 [cited 2026 Jan 27].

[11] Tricco AC, Khalil H, Holly C, Feyissa G, Godfrey C, Evans C, et al. Rapid reviews and the methodological rigor of evidence synthesis: a JBI position statement. JBI Evid Synth. 2022 Apr 1;20(4):944–9. 10.11124/JBIES-21-00371 35124684

[12] Abdala CVM. Digital databases of traditional, indigenous, complementary and integrative medicine: rapid review. [protocol]. Washington, DC: Center for Open Science; 2025. Available from: https://osf.io/ejfpa/ [cited 26 Feb 26].

[13] WHO global repository on traditional, complementary, and integrative medicine (TCIM) and indigenous knowledge (IK). Supplementary material, [online repository]. Meyrin: Zenodo; 2026. 10.5281/zenodo.18612754

[14] Ouzzani M, Hammady H, Fedorowicz Z, Elmagarmid A. Rayyan-a web and mobile app for systematic reviews. Syst Rev. 2016 Dec 5;5(1):210. 10.1186/s13643-016-0384-4 27919275 PMC5139140

[15] Sousa MSA, Wainwright M, Soares CB. Qualitative evidence synthesis: introductory guide. Bol Inst Saúde Est São Paulo. 2019;20(2):7–22. Available from: http://www.saude.sp.gov.br/resources/instituto-de-saude/homepage/bis/pdfs/bis_v20_n2_english.pdf [cited 2025 Feb 3].

[16] Ijaz N. What is traditional, complementary and integrative medicine: an operational typology. Amsterdam: SSRN; 2024. 10.2139/ssrn.4564463 41701581

[17] Parkhill A. Searching for the evidence: a practical guide to some online databases in chiropractic and osteopathy. Australas Chiropr Osteopathy. 2004 Nov;12(2):49–56. 17987213 PMC2051324

[18] Hussain N, Chanda R, Abir RA, Mou MA, Hasan MK, Ashraf MA. MPDB 2.0: a large scale and integrated medicinal plant database of Bangladesh. BMC Res Notes. 2021 Aug 6;14(1):301. 10.1186/s13104-021-05721-6 34362451 PMC8344187

[19] MPDB 2.0 [internet]. Dhaka: Tejgaon College, National University of Bangladesh; 2021. Available from: https://www.medicinalplantbd.com/ [cited 2025 Feb 28].

[20] ePlantLIBRA database [internet]. Brussels: European Food Information Resource; 2015. Available from: https://www.eurofir.org/our-tools/eplantlibra/ [cited 2025 Feb 28].

[21] Repositório saúde dos povos indígenas [internet]. Rio de Janeiro: Fiocruz; c1999. Available from: https://saudeindigena.fiocruz.br/ [cited 2025 Feb 28].

[22] Homeoindex [internet]. São Paulo: São Paulo Homeopathic Association; c1980. Available from: https://pesquisa.bvsalud.org/homeopatia/ [cited 2025 Feb 28].

[23] NuBBE database [internet]. São Paulo: São Paulo State University; 2013. Available from: http://nubbe.iq.unesp.br/portal/nubbedb.html [cited 2025 Feb 28].

[24] KNOW [internet]. Juneau: Oncology Association of Naturopathic Physicians; 2015. Available from: https://knowintegrativeoncology.org/ [cited 2025 Feb 28].

[25] Bioinformatics annotation database for molecular mechanism of traditional Chinese medicine (BATMAN-TCM) [internet]. Beijing: Beijing Institute of Lifeomics; 2016. Available from: http://bionet.ncpsb.org.cn/batman-tcm/#/home [cited 2025 Feb 28].

[26] CancerHSP [internet]. Shaanxi: Northwest A&F University; 2015. Available from: https://tcmsp-e.com/CancerHSP.php [cited 2025 Feb 28].

[27] Collective molecular activities of useful plants (CMAUP) [internet]. Shanghai: Fudan University; 2019. Available from: https://bidd.group/CMAUP/ [cited 2025 Feb 28].

[28] Database of constituents absorbed into the blood and metabolites of traditional Chinese medicines (DCABM-TCM) [internet]. Beijing: Beijing Proteome Research Center; 2023. Available from: http://bionet.ncpsb.org.cn/dcabm-tcm/#/Home [cited 2025 Feb 28].

[29] Encyclopedia of traditional Chinese medicine (ETCM) [internet]. Beijing: Institute of Chinese Materia Medica, China Academy of Chinese Medical Sciences; 2018. Available from: http://www.tcmip.cn/ETCM/ [cited 2025 Feb 28].

[30] 1 K medicinal plant genome database [internet]. Wuhan: Wuhan Benagen Tech Solutions Company Limited; 2021. Available from: http://www.herbgenome.com/ [cited 2025 Feb 28].

[31] Global pharmacopoeia genome database (GPGD) [internet]. Beijing: Institute of Chinese Materia Medica, China Academy of Chinese Medical Sciences; 2021. Available from: http://www.gpgenome.com [cited 2025 Feb 28].

[32] Herbal medicine omics database [internet]. Yan’an: Yan’an University, College of Life Sciences; 2017. Available from: http://herbalplant.ynau.edu.cn/ [cited 2025 Feb 28].

[33] Herbal ingredients’ targets (HIT) [internet]. Shanghai: Tongji University; 2011. Available from: http://www.badd-cao.net:2345/ [cited 2025 Feb 28].

[34] Medicinal materials DNA barcode database (MMDBD) [internet]. Sha Tin: Chinese University of Hong Kong; 2010. Available from: https://rdccm.cuhk.edu.hk/mherbsdb [cited 2025 Feb 28].

[35] Myrica rubra database [internet]. Hangzhou: ZheJiang Academy of Agricultural Sciences; 2020. Available from: http://www.bayberrybase.cn/ [cited 2025 Feb 28].

[36] Natural product activity and species source database (NPASS) [internet]. Shanghai: Fudan University; 2018. Available from: https://bidd.group/NPASS [cited 2025 Feb 28].

[37] DNA barcoding system for identifying herbal medicine [internet]. Beijing: Institute of Chinese Materia Medica, China Academy of Chinese Medical Sciences; c2013. Available from: http://www.tcmbarcode.cn/en/ [cited 2025 Feb 28].

[38] TIPdb [internet]. Kaohsiung City: Kaohsiung Medical University; 2013. Available from: http://cwtung.kmu.edu.tw/tipdb [cited 2025 Feb 28].

[39] Arctium lappa database [internet]. Dalian: School of Pharmacy, Liaoning University of Traditional Chinese Medicine; 2021. Available from: http://210.22.121.250:41352/ [cited 2025 Feb 28].

[40] ccTCM [internet]. Jiangsu: Nanjing University of Chinese Medicine; 2023. Available from: http://www.cctcm.org.cn [cited 2025 Feb 28].

[41] Chinese medicinal material images database [internet]. China Hong Kong SAR: Hong Kong Baptist University; 2013. Available from: https://library.hkbu.edu.hk/electronic/libdbs/mmd/index.html [cited 2025 Feb 28].

[42] Chinese medicine formulae images database [internet]. China Hong Kong SAR: Hong Kong Baptist University; 2018. Available from: https://library.hkbu.edu.hk/electronic/libdbs/cmfid/index.html [cited 2025 Feb 28].

[43] Chinese medicine resource net [internet]. China Hong Kong SAR: Hong Kong Baptist University; 2017. Available from: http://www.tcmdoc.cn [cited 2025 Feb 28].

[44] Chinese medicine specimen database [internet]. China Hong Kong SAR: Hong Kong Baptist University; 2013. Available from: https://library.hkbu.edu.hk/electronic/libdbs/scm_specimen.html [cited 2025 Feb 28].

[45] Drug herb interaction. DHI [internet]. Taipei: National Formosa University; 2007. Available from: https://drug-herb-interaction.netlify.app/ [cited 2025 Feb 28].

[46] HERB [internet]. Beijing: Beijing University of Chinese Medicine; 2020. Available from: http://herb.ac.cn/ [cited 2025 Feb 28].

[47] Integrated traditional Chinese medicine (ITCM). [internet]. Shanghai: Second Military Medical University; 2021. Available from: http://itcm.biotcm.net [cited 2025 Feb 28].

[48] SymMap [internet]. Beijing: Beijing University of Chinese Medicine; 2019. Available from: http://www.symmap.org/search [cited 2025 Feb 28].

[49] TCM Database@Taiwan [internet]. Taichung: China Medical University; 2011. Available from: http://tcm.cmu.edu.tw/index.php [cited 2025 Feb 28].

[50] TCM2COVID [internet]. Chengdu: Department of Bioinformatics, Chengdu University of Traditional Chinese Medicine; 2022. Available from: https://zhangy-lab.cn/tcm2covid/ [cited 2025 Feb 28].

[51] Traditional Chinese medicine on immuno-oncology (TCMIO) [internet]. Guangdong: Guangzhou University of Chinese Medicine; 2020. Available from: http://tcmio.xielab.net [cited 2025 Feb 28].

[52] TCMPG [internet]. Sichuan: Chengdu University of Traditional Chinese Medicine; 2021. Available from: https://cbcb.cdutcm.edu.cn/TCMPG/ [cited 2025 Feb 28].

[53] Traditional Chinese medicine database and analysis platform [internet]. Shaanxi: Northwest A&F University; 2014. Available from: https://tcmsp-e.com [cited 2025 Feb 28].

[54] Wanfang data, China medical collections [internet]. Beijing: Beijing Wanfang Data Co., Ltd; 2008. Available from: www.wanfangdata.com/medical/intr.asp [cited 2025 Feb 28].

[55] Chinese Herbal Dictionary [internet]. Complementary and Alternative Healing University; 2013. Available from: http://alternativehealing.org/Chinese_herbs_dictionary.htm [cited 2025 Feb 28].

[56] International traditional medicine clinical trial registry (ITMCTR) [internet]. Beijing: China Center for Evidence-Based Traditional Chinese Medicine; 2023. Available from: http://itmctr.ccebtcm.org.cn/en-US [cited 2025 Feb 28].

[57] ProBioQuest [internet]. Sha Tin: Chinese University of Hong Kong; 2022. Available from: http://kwanlab.bio.cuhk.edu.hk/PBQ/ [cited 2025 Feb 28].

[58] Egyptian herbal monograph [internet]. Cairo: Egyptian Drug Authority; 2020. Available from: http://eservices.edaegypt.gov.eg/Monograph/ [cited 2025 Feb 28].

[59] Ethiopian traditional medicine (ETM-DB) [internet]. Daejeon: Korea Advanced Institute of Science and Technology, Department of Bio and Brain Engineering, Bio-Synergy Research Center; 2019. Available from: https://biosoft.kaist.ac.kr/etm [cited 2025 Feb 28].

[60] Societas medicinale sinensis literature database [internet]. Munich: Societas Medicinae Sinensis; 2005. Available from: https://www.tcm.edu/mitgliederbereich/home.aspx [cited 2025 Feb 28].

[61] Database of Unani medicine [internet]. Bochum: Ruhr-Universität Bochum; 2025. Available from: https://dbs-lin.ruhr-uni-bochum.de/unianimedizin/ [cited 2025 Feb 28].

[62] Arzneipflanzenlexikon [internet]. Bonn: Kooperation Phytopharmaka; 2015. Available from: https://arzneipflanzenlexikon.info/ [cited 2025 Feb 28].

[63] ARTHEDATA [internet]. Ottersberg: Ottersberg University of Applied Sciences; 2009. Available from: https://cambase.uni-wh.de/arthedata/ [cited 2025 Feb 28].

[64] Homeopathy basic research experiments database (HomBRex) [internet]. Witten: Carstens-Stiftung; 2002. Available from: https://www.carstens-stiftung.de/datenbanken-zur-integrativen-medizin.html#/ [cited 2025 Feb 28].

[65] Veterinary clinical research database in homeopathy (HomVetCR) [internet]. Witten: Carstens-Stiftung; 2006. Available from: https://www.carstens-stiftung.de/ [cited 2025 Feb 28].

[66] Veterinary clinical research database for homeopathy (VetCR) [internet]. Essen: Carstens-Stiftung; 2018. Available from: https://www.carstens-stiftung.de/datenbanken-zur-integrativen-medizin.html#/login [cited 2025 Feb 28].

[67] Complementary and alternative medicine database (CAMbase) [internet]. Witten: Universidade de Witten/Herdecke; 2007. Available from: https://cambase.uni-wh.de/camdb/de/ [cited 2025 Feb 28].

[68] ANPDB. African natural products database [internet]. Freiburg: University of Freiburg; 2017. Available from: https://african-compounds.org/anpdb/ [cited 2025 Feb 28].

[69] SuperNatural 3.0 [internet]. Berlin: Institute of Physiology, Charité, Universitätsmedizin Berlin; 2022. Available from: https://bioinf-applied.charite.de/supernatural_3/ [cited 2025 Feb 28].

[70] OSTLIB [internet]. Siegen: Instituto de Estudos Osteopáticos; 2022. Available from: https://ostlib.de/ [cited 2025 Feb 28].

[71] DATADIWAN [internet]. Berlin: Bernhard Harrer Knowledge Transfer; 1995. Available from: https://www.datadiwan.de/suche/index_e.htm?dws000e_.htm [cited 2025 Feb 28].

[72] AMMPDB VER 1.1. Anti Mtb medicinal plants database [internet]. New Delhi: Jawaharlal Nehru University; 2023. Available from: https://www.ammpdb.com/ [cited 2025 Feb 28].

[73] Āyurveda formulation advanced database [internet]. Maharashtra: National Library of Ayurved Medicine; 2009. Available from: https://nlam.in/search_module.php [cited 2025 Feb 28].

[74] Digital helpline for ayurveda research articles (DHARA) [internet]. Tamil Nadu: AVT Institute for Advanced Research; 2010. Available from: http://www.dharaonline.org/Forms/Home.aspx [cited 2025 Feb 28].

[75] Global online ayurveda library (GOAL) [internet]. Edison: The Santhigram Foundation; 2023. Available from: https://ayurvedalibrary.org/ [cited 2025 Feb 28].

[76] Ayush research portal [internet]. Telangana: National Institute of Indian Medical Heritage; 2011. Available from: https://ayushportal.nic.in/ [cited 2025 Feb 28].

[77] Indian medicinal plants database [internet]. Bengaluru: National Medicinal Plants Board; c1993. Available from: https://www.medicinalplants.in/ [cited 2025 Feb 28].

[78] AromaDb [internet]. New Delhi: Central Institute of Medicinal and Aromatic Plants; 2018. Available from: https://aromadb.cimapbioinfo.in/ [cited 2025 Jan 31].

[79] DISPEL. Diseases plants eliminate [internet]. Roorkee: Indian Institute of Technology Roorkee; 2023. Available from: https://compbio.iitr.ac.in/dispel/ [cited 2025 Feb 28].

[80] Indian Medicinal Plants. Phytochemistry and therapeutics (IMPPAT) [internet]. Tamil Nadu: The Institute of Mathematical Sciences; 2018. Available from: https://cb.imsc.res.in/imppat [cited 2025 Feb 28].

[81] North East India medicinal plants database (NEI-MPDB) [internet]. Jorhat, Assam: North East Institute of Science and Technology, Council of Scientific & Industrial Research; 2022. Available from: https://neist.res.in/neimpdb/ [cited 2025 Feb 28].

[82] PlantPepDB [internet]. New Delhi: National Institute of Plant Genome Research; 2020. Available from: http://14.139.61.8/PlantPepDB/index.php [cited 2025 Feb 28].

[83] Prediction tool for plant-derived antimicrobial peptides (PTPAMP) [internet]. New Delhi: National Institute of Plant Genome Research; 2022. Available from: http://www.nipgr.ac.in/PTPAMP/ [cited 2025 Feb 28].

[84] Anti-Mtb medicinal plant database [internet]. New Delhi: School of Environmental Sciences, Jawaharlal Nehru University; 2023. Available from: https://www.ammpdb.com/ [cited 2025 Feb 28].

[85] Indian medicinal plants nomenclature database [internet]. Bengaluru: Foundation for Revitalisation of Local Health Traditions, University of Trans-Disciplinary Health Sciences and Technology; c1993. Available from: https://envis.frlht.org/implad [cited 2025 Feb 28].

[86] Traded medicinal plant database [internet]. New Delhi, National Medicinal Plant Board of India; 2020. Available from: https://tradedmedicinalplants.org/ [cited 2025 Feb 28].

[87] EIACP resource partner on biodiversity [internet]. Kolkata: Botanical Survey of India; 1996. Available from: http://bsienvis.nic.in/Database/MedicinalPlants_3939.aspx [cited 2025 Feb 28].

[88] Naturally occurring plant based anticancerous compound-activity-target database (NPACT) [internet]. Noida: Institute of Cytology and Preventive Oncology; 2012. Available from: http://crdd.osdd.net/raghava/npact/ [cited 2025 Feb 28].

[89] MeFSAT. Medicinal fungi secondary metabolites and therapeutics [internet]. Tamil Nadu: The Institute of Mathematical Sciences; 2021. Available from: https://cb.imsc.res.in/mefsat/ [cited 2025 Feb 28].

[90] Seaweed metabolite database [internet]. Tamil Nadu: Davis & Vasanthi, Bioinformation; 2011. Available from: http://www.swmd.co.in/ [cited 2025 Feb 28]. 10.6026/97320630005361 PMC3053594 21423723

[91] UNaProd [internet]. Tehran: School of Persian Medicine, Tehran University of Medical Sciences; 2020. Available from: https://unaprod.com/ [cited 2025 Feb 28].

[92] KampoDB [internet]. Toyama: University of Toyama; 2021. Available from: https://www.inm.u-toyama.ac.jp/en/database/ [cited 2025 Feb 28].

[93] ETHMEDmmm [internet]. Toyama: University of Toyama; 2022. Available from: https://ethmed.toyama-wakan.net/SearchEn/ [cited 2025 Feb 28].

[94] TradMPD [internet]. Toyama: University of Toyama; 2014. Available from: https://dentomed.toyama-wakan.net/en/ [cited 2025 Feb 28].

[95] Global information hub on integrative medicine (GlobInMed) [internet]. Shah Alam: Ministry of Health, Institute for Medical Research; 2003. Available from: https://globinmed.com/ [cited 2025 Feb 28].

[96] New Zealand’s Māori centre of research excellence (NPM) [internet]. Aotearoa: The University of Auckland; 2002. Available from: https://www.maramatanga.ac.nz/ [cited 2025 Feb 28].

[97] Complementary and alternative medicine for cancer (CAM Cancer) [internet]. Tromsø: National Research Center in Complementary and Alternative Medicine, Arctic University of Norway; 2007. Available from: https://cam-cancer.org/ [cited 2025 Feb 28].

[98] AntiViral phyto chemical database (AVPCD) [internet]. Takht Bhai: Shahid Khan Lab; 2023. Available from: https://avpcd.habdsk.org/ [cited 2025 Feb 28].

[99] Philippine traditional knowledge digital library on health [internet].Manila: Philippine Council for Health Research and Development, Philippine Institute of Traditional and Alternative Health Care and the University of the Philippines Manila; 2011. Available from: https://www.tkdlph.com/index.php [cited 2025 Feb 28].

[100] TM-MC 2.0 [internet]. Daejeon: Korea Institute of Oriental Medicine; 2024. Available from: https://tm-mc.kr [cited 2025 Feb 28].

[101] PharmDB-K [internet]. Seoul: Seoul National University; 2015. Available from: http://pharmdb-k.org [cited 2025 Feb 28].

[102] PlantMolecularTasteDB [internet]. Bucharest: Carol Davila University of Medicine and Pharmacy; 2022. Available from: http://www.plantmoleculartastedb.org/index.php [cited 2025 Feb 28].

[103] The Phyto4Health [internet]. Moscow: Institute of Biomedical Chemistry; 2023. Available from: http://www.way2drug.com/p4h/ [cited 2025 Feb 28].

[104] Traditional Chinese medicine information database (TCM-ID) [internet]. Singapore: National University of Singapore; 2006. Available from: https://bidd.group/TCMID/ [cited 2025 Feb 28].

[105] South African natural compounds database (SANCDB) [internet]. Grahamstown: Rhodes University; 2015. Available from: https://sancdb.rubi.ru.ac.za [cited 2025 Feb 28].

[106] CANNUSE [internet]. Barcelona: Institut Botànic de Barcelona; 2021. Available from: http://cannusedb.csic.es [cited 2025 Feb 28].

[107] Natural Products ^13^C NMR Database (NAPROC-13). [internet]. Salamanca: Universidad d Salamanca; 2007. Available from: http://c13.usal.es [cited 2025 Feb 28].

[108] Mistletoe-therapy [internet]. Dornach: School of Spiritual Science; c2008. Available from: https://www.mistletoe-therapy.org/ [cited 2025 Feb 28].

[109] Herbal medicine database, Thaicrudedrug [internet]. Ubon Ratchathani: Faculty of Pharmaceutical Sciences, Ubon Ratchathani University; 2012. Available from: https://ayushportal.nic.in/ [ cited 2025 Feb 28].

[110] Thai medicinal plant recipe database (MANOSROI III) [internet]. Chiang Mai: Manosé Research Center; 2013. Available from: https://www.manose.co/portfolio-item/work-2/ [cited 2025 Feb 28].

[111] Electroacupuncture. Clinical studies database [internet]. London: Churchill Livingstone; 2007. Available from: http://www.electroacupunctureknowledge.com/mayor_database/home.htm [cited 2025 Feb 28].

[112] Clinical outcome research in homeopathy database (CORE-Hom) [internet]. London: Homeopathy Research Institute; 2014. Available from: https://www.hri-research.org/resources/research-databases/homeopathy-research-databases/ [cited 2025 Feb 28].

[113] Yoga therapy library [internet]. London: Yoga Therapy Library; 2022. Available from: https://www.yogatherapylibrary.com/ [cited 2025 Feb 28].

[114] About herbs, botanicals & other products [internet]. New York: Memorial Sloan Kettering Cancer Center; 2002. Available from: https://www.mskcc.org/cancer-care/diagnosis-treatment/symptom-management/integrative-medicine/herbs [cited 2025 Feb 28].

[115] Herbal medicine compendium [internet]. Rockville: U.S. Pharmacopeial Convention; 2013. Available from: https://hmc.usp.org/ [cited 2025 Feb 28].

[116] HerbMed & HerbMedPro [internet]. Austin: American Botanical Council; 1998. Available from: www.herbmed.org [cited 2025 Feb 28].

[117] Medline plus. Herbs and supplements [internet]. Maryland: US National Library of Medicine; 2005. Available from: https://medlineplus.gov/druginfo/herb_All.html [cited 2025 Feb 28].

[118] SUPP. Al. Discover supplement-drug interactions [internet]. Seattle: The Allen Institute for Artificial Intelligence; 2020. Available from: https://supp.ai/ [cited 2025 Feb 28].

[119] AcuTrials® [internet]. Portland: Oregon College of Oriental Medicine (OCOM); 2025. Available from: https://acutrials.ocom.edu/ [cited 2025 Feb 28].

[120] Qigong and energy medicine database™ [internet]. Los Altos: Qigong Institute; c1990. Available from: https://www.qigonginstitute.org/abstracts [cited 2025 Feb 28].

[121] Cochrane complementary medicine [internet]. Washington, DC: Georgetown University; 1996. Available from: https://cam.cochrane.org/ [cited 2025 Feb 28].

[122] Native American ethnobotany [internet]. Fort Worth: Botanical Research Institute of Texas; 1977. Available from: http://naeb.brit.org/ [cited 2025 Feb 28].

[123] Native health database [internet]. Albuquerque: University of New Mexico; c1990. Available from: https://nativehealthdatabase.net/ [cited 2025 Feb 28].

[124] AnthroMed [internet]. Moab: Physicians’ Association for Anthroposophic Medicine; 2024. Available from: https://www.anthromed.org/ [cited 2025 Feb 28].

[125] Allied and complementary medicine database [internet]. Ipswich: EBSCO; 1985. Available from: https://www.ebsco.com/products/research-databases/allied-and-complementary-medicine-database-amed [cited 2025 Feb 28].

[126] NatMed Pro [internet]. Stockton: Therapeutic Research Center; 2025. Available from: https://trchealthcare.com/product/natmed-pro/ [cited 2025 Feb 28].

[127] Computer access to research on dietary supplements (CARDS) [internet]. Bethesda: National Institutes of Health; 2021. Available from: https://cards.od.nih.gov/ [cited 2025 Feb 28].

[128] Dietary supplements compendium (DSC) [internet]. Rockville: U.S. Pharmacopeia; 2009. Available from: https://www.usp.org/products/dietary-supplements-compendium [cited 2025 Feb 28].

[129] DSID. Dietary supplement database [internet]. Bethesda: National Institutes of Health; 2009. Available from: https://dietarysupplementdatabase.usda.nih.gov/ [cited 2025 Feb 28].

[130] Dietary supplement label database (DSLD) [internet]. Bethesda: National Institutes of Health; 2013. Available from: https://ods.od.nih.gov/Research/Dietary_Supplement_Label_Database.aspx [cited 2025 Feb 28].

[131] The national cancer institute natural products repository [internet]. Bethesda: National Cancer Institute; 1986. Available from: https://dtp.cancer.gov/organization/npb/introduction.htm [cited 2025 Feb 28].

[132] Antimicrobial peptide database. APD [internet]. Omaha: University of Nebraska; 2017. Available from: https://aps.unmc.edu/ [cited 2025 Feb 28].

[133] Lexi-natural products [internet]. Waltham: Wolters Kluwer; 1996. Available from: http://webstore.lexi.com/Store/PDA-Software-for-Dentists/Lexi-Natural-Products [cited 2025 Feb 28].

[134] Natural product–drug interaction research database (NaPDI) [internet]. Spokane: Center of Excellence for Natural Product-Drug Interaction Research; 2015. Available from: https://repo.napdi.org/ [cited 2025 Feb 28].

[135] NP-MRD. [internet]. Alberta: University of Alberta; 2022. Available from: https://np-mrd.org [cited 2025 Feb 28].

[136] Osteopathic medical digital repository. (OSTMED.DR) [internet]. Blacksburg: Edward Via College of Osteopathic Medicine; 2006. Available from: https://ostemed-dr.contentdm.oclc.org/ [cited 2025 Feb 28].

[137] VIETHERB [internet]. Ho Chi Minh: Ho Chi Minh International University; 2016. Available from: https://vietherb.com.vn/ [cited 2025 Feb 28].

[138] Program of applied research to popular medicine in the Caribbean (TRAMIL) [internet]. Santo Domingo: TRAMIL Network; 1982. Available from: https://tramil.net/en [cited 2025 Nov 10].

[139] MOSAIC [internet]. São Paulo: TCIM Network of Americas; 2018. Available from: mtci.bvsalud.org [cited 2025 Feb 28].

[140] Marx BL, Milley R, Cantor DG, Ackerman DL, Hammerschlag R. AcuTrials®: an online database of randomized controlled trials and systematic reviews of acupuncture. BMC Complement Altern Med. 2013 Jul 19;13(1):181. 10.1186/1472-6882-13-181 23866767 PMC3750563

[141] Alt HealthWatch [internet]. Ipswich: EBSCO Information Services; 2001. Available from: https://www.ebsco.com/products/research-databases/alt-healthwatch [cited 2025 Feb 28].

[142] Vardell E. AMED: The allied and complementary medicine database. Med Ref Serv Q. 2016 Oct-Dec;35(4):434–9. 10.1080/02763869.2016.1220759 27657370

[143] Boehm K, Raak C, Vollmar HC, Ostermann T. An overview of 45 published database resources for complementary and alternative medicine. Health Info Libr J. 2010 Jun;27(2):93–105. 10.1111/j.1471-1842.2010.00888.x 20565550

[144] Kanneganti J, Mina U, Singh A, Gautam A, Somvanshi P. Anti Mtb Medicinal Plants Database (AMMPDB): a curated database of Indian anti-tubercular medicinal plants. J Ayurveda Integr Med. 2023 Mar-Apr;14(2):100712. 10.1016/j.jaim.2023.100712 37120901 PMC10172712

[145] Li XL, Zhang JQ, Shen XJ, Zhang Y, Guo DA. Overview and limitations of database in global traditional medicines: a narrative review. Acta Pharmacol Sin. 2025 Feb;46(2):235–63. 10.1038/s41401-024-01353-1 39095509 PMC11747326

[146] Kumar Y, Prakash O, Tripathi H, Tandon S, Gupta MM, Rahman LU, et al. AromaDb: a database of medicinal and aromatic plant’s aroma molecules with phytochemistry and therapeutic potentials. Front Plant Sci. 2018 Aug 13;9:1081. 10.3389/fpls.2018.01081 30150996 PMC6099104

[147] Wegener T, Nieber K, Kraft K, Siegmund S, Kelber O, Jobst D, et al. Versorgungsforschung mit pflanzlichen Arzneimitteln – Die pharmako-epidemiologische Datenbank PhytoVIS. Z Phytother. 2021 Jun 16;42(3):127–35. German. 10.1055/a-1406-4253

[148] Ullah S, Rahman W, Ullah F, Ullah A, Ahmad G, Ijaz M, et al. AVPCD: a plant-derived medicine database of antiviral phytochemicals for cancer, Covid-19, malaria and HIV. Database (Oxford). 2023 Aug 18;2023:baad056. 10.1093/database/baad056 37594855 PMC10437090

[149] Ostermann T, Zillmann H, Matthiessen PF. [CAMbase–the realisation of an XML-based bibliographical database system for complementary and alternative medicine]. Z Arztl Fortbild Qualitatssich. 2004 Sep;98(6):501–7. German. 15527194

[150] Ostermann T, Zillmann H, Raak CK, Buessing A, Matthiessen PF. CAMbase–a XML-based bibliographical database on complementary and alternative medicine (CAM). Biomed Digit Libr. 2007 Apr 3;4(1):2. 10.1186/1742-5581-4-2 17407592 PMC1853104

[151] Tao W, Li B, Gao S, Bai Y, Shar PA, Zhang W, et al. CancerHSP: anticancer herbs database of systems pharmacology. Sci Rep. 2015 Jun 15;5(1):11481. 10.1038/srep11481 26074488 PMC4466901

[152] Balant M, Gras A, Ruz M, Vallès J, Vitales D, Garnatje T. Traditional uses of cannabis: an analysis of the CANNUSE database. J Ethnopharmacol. 2021 Oct 28;279:114362. 10.1016/j.jep.2021.114362 34171396

[153] Balant M, Gras A, Gálvez F, Garnatje T, Vallès J, Vitales D. CANNUSE, a database of traditional cannabis uses – an opportunity for new research. Database (Oxford). 2021 May 1;2021:baab024. 10.1093/database/baab024 33942873 PMC8087868

[154] Haggans CJ, Regan KS, Brown LM, Wang C, Krebs-Smith J, Coates PM, et al. Computer access to research on dietary supplements: a database of federally funded dietary supplement research. J Nutr. 2005 Jul;135(7):1796–9. 10.1093/jn/135.7.1796 15987867

[155] Yang D, Zhu Z, Yao Q, Chen C, Chen F, Gu L, et al. ccTCM: a quantitative component and compound platform for promoting the research of traditional Chinese medicine. Comput Struct Biotechnol J. 2023 Nov 20;21:5807–17. 10.1016/j.csbj.2023.11.030 38213899 PMC10781882

[156] Zeng X, Zhang P, Wang Y, Qin C, Chen S, He W, et al. CMAUP: a database of collective molecular activities of useful plants. Nucleic Acids Res. 2019 Jan 8;47 D1:D1118–27. 10.1093/nar/gky965 30357356 PMC6324012

[157] Clausen J, Moss S, Tournier A, Lüdtke R, Albrecht H. CORE-Hom: a powerful and exhaustive database of clinical trials in homeopathy. Homeopathy. 2014 Oct;103(4):219–23. 10.1016/j.homp.2014.07.001 25439037

[158] Liu X, Liu J, Fu B, Chen R, Jiang J, Chen H, et al. DCABM-TCM: a database of constituents absorbed into the blood and metabolites of traditional Chinese medicine. J Chem Inf Model. 2023 Aug 14;63(15):4948–59. 10.1021/acs.jcim.3c00365 37486750 PMC10428213

[159] Manosroi A, Akazawa H, Pattamapun K, Kitdamrongtham W, Akihisa T, Manosroi W, et al. Potent anti-proliferative effects against oral and cervical cancers of Thai medicinal plants selected from the Thai/Lanna medicinal plant recipe database “MANOSROI III”. Pharm Biol. 2015 Jul;53(7):1075–81. 10.3109/13880209.2014.959613 25612774

[160] Manosroi A, Tangjai T, Chankhampan C, Manosroi W, Najarut Y, Kitdamrongtham W, et al. Potent phosphodiesterase inhibition and nitric oxide release stimulation of anti‐impotence Thai medicinal plants from “MANOSROI III” database. Evid Based Complement Alternat Med. 2017 Jan 25;2017:9806976. 10.1155/2017/9806976 28811831 PMC5547717

[161] Manosroi A, Lohcharoenkal W, Khonsung P, Manosroi W, Manosroi J. Potent antihypertensive activity of Thai-Lanna medicinal plants and recipes from “MANOSROI III” database. Pharm Biol. 2013 Nov;51(11):1426–34. 10.3109/13880209.2013.796391 23869399

[162] Sangma C, Chuakheaw D, Jongkon N, Saenbandit K, Nunrium P, Uthayopas P, et al. Virtual screening for anti-HIV-1 RT and anti-HIV-1 PR inhibitors from the Thai medicinal plants database: a combined docking with neural networks approach. Comb Chem High Throughput Screen. 2005 Aug;8(5):417–29. 10.2174/1386207054546469 16101581

[163] Wu CS, Chen YH, Chen CL, Chien SK, Syifa N, Hung YC, et al. Constructing a bilingual website with validated database for Herb and Western medicine interactions using Ginseng, Ginkgo and Dong Quai as examples. BMC Complement Altern Med. 2019 Nov 27;19(1):335. 10.1186/s12906-019-2731-1 31775730 PMC6881993

[164] Singh K, Maurya H, Singh P, Panda P, Behera AK, Jamal A, et al. DISPEL: database for ascertaining the best medicinal plants to cure human diseases. Database (Oxford). 2023 Oct 16;2023:baad073. 10.1093/database/baad073 37847815 PMC10581335

[165] Wong TH, But GWC, Wu HY, Tsang SSK, Lau DTW, Shaw PC. Medicinal Materials DNA Barcode Database (MMDBD) version 1.5-one-stop solution for storage, BLAST, alignment and primer design. Database (Oxford). 2018 Jan 1;2018:2018. 10.1093/database/bay112 30335153 PMC6193215

[166] Andrews KW, Roseland JM, Gusev PA, Palachuvattil J, Dang PT, Savarala S, et al. Analytical ingredient content and variability of adult multivitamin/mineral products: national estimates for the dietary supplement ingredient database. Am J Clin Nutr. 2017 Feb;105(2):526–39. 10.3945/ajcn.116.134544 27974309 PMC5267296

[167] Dwyer JT, Picciano MF, Betz JM, Fisher KD, Saldanha LG, Yetley EA, et al. Progress in developing analytical and label-based dietary supplement databases at the NIH Office of Dietary Supplements. J Food Compos Anal. 2008 Feb;21:S83–93. 10.1016/j.jfca.2007.07.010 25346570 PMC4208495

[168] Plumb J, Lyons J, Nørby K, Thomas M, Nørby E, Poms R, et al.; PlantLIBRA Consortia. ePlantLIBRA: a composition and biological activity database for bioactive compounds in plant food supplements. Food Chem. 2016 Feb 15;193:121–7. 10.1016/j.foodchem.2015.03.126 26433297

[169] Su W, Liao M, Tan H, Chen Y, Zhao R, Jin W, et al. Identification of autophagic target RAB13 with small-molecule inhibitor in low-grade glioma via integrated multi-omics approaches coupled with virtual screening of traditional Chinese medicine databases. Cell Prolif. 2021 Dec;54(12):e13135. 10.1111/cpr.13135 34632655 PMC8666277

[170] Li X, Ren J, Zhang W, Zhang Z, Yu J, Wu J, et al. LTM-TCM: a comprehensive database for the linking of traditional Chinese medicine with modern medicine at molecular and phenotypic levels. Pharmacol Res. 2022 Apr;178:106185. 10.1016/j.phrs.2022.106185 35306140

[171] Bultum LE, Woyessa AM, Lee D. ETM-DB: integrated Ethiopian traditional herbal medicine and phytochemicals database. BMC Complement Altern Med. 2019 Aug 14;19(1):212. 10.1186/s12906-019-2634-1 31412866 PMC6692943

[172] Su X, Yang L, Wang D, Shu Z, Yang Y, Chen S, et al. 1 K medicinal plant genome database: an integrated database combining genomes and metabolites of medicinal plants. Hortic Res. 2022 Mar 23;9:uhac075. 10.1093/hr/uhac075 35669712 PMC9160725

[173] Fang S, Dong L, Liu L, Guo J, Zhao L, Zhang J, et al. HERB: a high-throughput experiment- and reference-guided database of traditional Chinese medicine. Nucleic Acids Res. 2021 Jan 8;49 D1:D1197–206. 10.1093/nar/gkaa1063 33264402 PMC7779036

[174] Wang X, Zhang J, He S, Gao Y, Ma X, Gao Y, et al. HMOD: an omics database for herbal medicine plants. Mol Plant. 2018 May 7;11(5):757–9. 10.1016/j.molp.2018.03.002 29524650

[175] Kang H, Tang K, Liu Q, Sun Y, Huang Q, Zhu R, et al. HIM-herbal ingredients in-vivo metabolism database. J Cheminform. 2013 May 31;5(1):28. 10.1186/1758-2946-5-28 23721660 PMC3679852

[176] May BH, Deng S, Zhang AL, Lu C, Xue CCL. In silico database screening of potential targets and pathways of compounds contained in plants used for psoriasis vulgaris. Arch Dermatol Res. 2015 Sep;307(7):645–57. 10.1007/s00403-015-1577-8 26142738

[177] Van Wijk R, Albrecht H. Proving and therapeutic experiments in the HomBRex basic homeopathy research database. Homeopathy. 2007 Oct;96(4):252–7. 10.1016/j.homp.2007.08.007 17954383

[178] Mesquita A, Martins CC, Cepeda LMR. Homeoindex: new computerized bibliographical database of homoeopathic literature. Br Homeopath J. 1994 Oct 10;83(4):209–15. 10.1016/S0007-0785(05)80794-X

[179] Clausen J, Albrecht H. Database on veterinary clinical research in homeopathy. Homeopathy. 2010 Jul;99(3):189–91. 10.1016/j.homp.2010.03.005 20674843

[180] Index to chiropractic literature ICL [internet]. Arlington: Association of Chiropractic Colleges; 1980. Available from: https://www.chiroindex.org/#results [cited 2025 Feb 28].

[181] Mohanraj K, Karthikeyan BS, Vivek-Ananth RP, Chand RPB, Aparna SR, Mangalapandi P, et al. IMPPAT: a curated database of Indian medicinal plants, phytochemistry and therapeutics. Sci Rep. 2018 Mar 12;8(1):4329. 10.1038/s41598-018-22631-z 29531263 PMC5847565

[182] Green J, Wright H, Seely D, Legacy M, Anderson M, Armstrong H, et al. A survey of multidisciplinary healthcare providers utilizing the KNOWintegrativeoncology.org educational platform. BMC Complement Med Ther. 2022 Apr 28;22(1):118. 10.1186/s12906-022-03601-5 35484545 PMC9047465

[183] Allais G, Voghera D, De Lorenzo C, Mana O, Benedetto C. Access to databases in complementary medicine. J Altern Complement Med. 2000 Jun;6(3):265–74. 10.1089/acm.2000.6.265 10890337

[184] Clauson KA, Peak AS, Marsh WA, DiScala S, Bellinger RR. Clinical decision support tools: focus on dietary supplement databases. Altern Ther Health Med. 2008 May-Jun;14(3):36–40. 18517104

[185] Manosroi J, Sainakham M, Manosroi W, Manosroi A. Anti-proliferative and apoptosis induction activities of extracts from Thai medicinal plant recipes selected from MANOSROI II database. J Ethnopharmacol. 2012 May 7;141(1):451–9. 10.1016/j.jep.2012.03.010 22440260

[186] Manosroi J, Moses ZZ, Manosroi W, Manosroi A. Hypoglycemic activity of Thai medicinal plants selected from the Thai/Lanna Medicinal Recipe Database MANOSROI II. J Ethnopharmacol. 2011 Oct 31;138(1):92–8. 10.1016/j.jep.2011.08.049 21925259

[187] Manosroi A, Akazawa H, Akihisa T, Jantrawut P, Kitdamrongtham W, Manosroi W, et al. In vitro anti-proliferative activity on colon cancer cell line (HT-29) of Thai medicinal plants selected from Thai/Lanna medicinal plant recipe database “MANOSROI III”. J Ethnopharmacol. 2015 Feb 23;161:11–7. 10.1016/j.jep.2014.11.038 25481081

[188] Kitdamrongtham W, Manosroi A, Akazawa H, Gidado A, Stienrut P, Manosroi W, et al. Potent anti-cervical cancer activity: synergistic effects of Thai medicinal plants in recipe N040 selected from the MANOSROI III database. J Ethnopharmacol. 2013 Aug 26;149(1):288–96. 10.1016/j.jep.2013.06.037 23831080

[189] Vivek-Ananth RP, Sahoo AK, Kumaravel K, Mohanraj K, Samal A. MeFSAT: a curated natural product database specific to secondary metabolites of medicinal fungi. RSC Adv. 2021 Jan 12;11(5):2596–607. 10.1039/D0RA10322E 35424258 PMC8693784

[190] Ashraf MA, Khatun A, Sharmin T, Mobin F, Tanu AR, Morshed T, et al. MPDB 1.0: a medicinal plant database of Bangladesh. Bioinformation. 2014 Jun 30;10(6):384–6. 10.6026/97320630010384 25097384 PMC4110432

[191] Ren H, He Y, Qi X, Zheng X, Zhang S, Yu Z, et al. The bayberry database: a multiomic database for Myrica rubra, an important fruit tree with medicinal value. BMC Plant Biol. 2021 Oct 6;21(1):452. 10.1186/s12870-021-03232-x 34615485 PMC8493685

[192] Birer-Williams C, Gufford BT, Chou E, Alilio M, VanAlstine S, Morley RE, et al. A new data repository for pharmacokinetic natural product-drug interactions: from chemical characterization to clinical studies. Drug Metab Dispos. 2020 Oct;48(10):1104–12. 10.1124/dmd.120.000054 32601103 PMC7543481

[193] Kiewhuo K, Gogoi D, Mahanta HJ, Rawal RK, Das D, Sastry GN. North East India medicinal plants database (NEI-MPDB). Comput Biol Chem. 2022 Oct;100:107728. 10.1016/j.compbiolchem.2022.107728 35952423

[194] Franke H. OSTLIB – Die osteopathische datenbank. Osteopath Med. 2022;23(3):40. German. 10.1016/S1615-9071(22)00086-7

[195] Fitterling L, Powers E, Vardell E. OSTMED.DR®, an osteopathic medicine digital library. Med Ref Serv Q. 2018 Jan-Mar;37(1):74–80. 10.1080/02763869.2018.1404390 29327987

[196] Lee JH, Park KM, Han DJ, Bang NY, Kim DH, Na H, et al. PharmDB-K: integrated bio-pharmacological network database for traditional Korean medicine. PLoS One. 2015 Nov 10;10(11):e0142624. 10.1371/journal.pone.0142624 26555441 PMC4640719

[197] Chan PL, Lauw S, Ma KL, Kei N, Ma KL, Wong YO, et al. ProBioQuest: a database and semantic analysis engine for literature, clinical trials and patents related to probiotics. Database (Oxford). 2022 Jul 15;2022:baac059. 10.1093/database/baac059 35849028 PMC9290863

[198] Wootton JC. Directory of databases for research into alternative and complementary medicine: an update. J Altern Complement Med. 1997 Winter;3(4):401–3. 10.1089/acm.1997.3.401 9449062

[199] Wu Y, Zhang F, Yang K, Fang S, Bu D, Li H, et al. SymMap: an integrative database of traditional Chinese medicine enhanced by symptom mapping. Nucleic Acids Res. 2019 Jan 8;47 D1:D1110–7. 10.1093/nar/gky1021 30380087 PMC6323958

[200] Chen HY, Chang SS, Chan YC, Chen CY. Discovery of novel insomnia leads from screening traditional Chinese medicine database. J Biomol Struct Dyn. 2014;32(5):776–91. 10.1080/07391102.2013.790849 23730798

[201] Yang SC, Chang SS, Chen HY, Chen CYC. Identification of potent EGFR inhibitors from TCM Database@Taiwan. PLOS Comput Biol. 2011 Oct;7(10):e1002189. 10.1371/journal.pcbi.1002189 22022246 PMC3192800

[202] Gao H. Predicting tyrosinase inhibition by 3D QSAR pharmacophore models and designing potential tyrosinase inhibitors from traditional Chinese medicine database. Phytomedicine. 2018 Jan 1;38:145–57. 10.1016/j.phymed.2017.11.012 29425647

[203] Chen CYC. TCM Database@Taiwan: the world’s largest traditional Chinese medicine database for drug screening in silico. PLoS One. 2011 Jan 6;6(1):e15939. 10.1371/journal.pone.0015939 21253603 PMC3017089

[204] Wu CW, Chen HY, Yang CW, Chen YC. Deciphering the efficacy and mechanisms of Chinese herbal medicine for diabetic kidney disease by integrating web-based biochemical databases and real-world clinical data: retrospective cohort study. JMIR Med Inform. 2021 May 11;9(5):e27614. 10.2196/27614 33973855 PMC8150407

[205] Chen C, Zhang R, Wei W, Zhou J. Anti-COVID-19 traditional Chinese medicine database. Coronaviruses. 2022 Dec;3(6). 10.2174/2666796704666221028151334

[206] Ren L, Xu Y, Ning L, Pan X, Li Y, Zhao Q, et al. TCM2COVID: a resource of anti-COVID-19 traditional Chinese medicine with effects and mechanisms. iMeta. 2022 Aug 5;1(4):e42. 10.1002/imt2.42 36245702 PMC9537919

[207] TCMBank [internet]. Guangdong: Artificial Intelligence Medical Center, School of Intelligent Systems Engineering, Sun Yat-sen University; 2019. Available from: https://tcmbank.cn/ [cited 2025 Feb 28].

[208] Chen X, Zhou H, Liu YB, Wang JF, Li H, Ung CY, et al. Database of traditional Chinese medicine and its application to studies of mechanism and to prescription validation. Br J Pharmacol. 2006 Dec;149(8):1092–103. 10.1038/sj.bjp.0706945 17088869 PMC2014641

[209] Liu Z, Cai C, Du J, Liu B, Cui L, Fan X, et al. TCMIO: a comprehensive database of traditional Chinese medicine on immuno-oncology. Front Pharmacol. 2020 Apr 15;11:439. 10.3389/fphar.2020.00439 32351388 PMC7174671

[210] Ionov N, Druzhilovskiy D, Filimonov D, Poroikov V. Phyto4Health: database of phytocomponents from Russian pharmacopoeia plants. J Chem Inf Model. 2023 Apr 10;63(7):1847–51. 10.1021/acs.jcim.2c01567 36995916

[211] Fredriksson M. India’s traditional knowledge digital library and the politics of patent classifications. Law Crit. 2023;34(1):1–19. 10.1007/s10978-021-09299-7 36915708 PMC9999701

[212] Kim SK, Lee MK, Jang H, Lee JJ, Lee S, Jang Y, et al. TM-MC 2.0: an enhanced chemical database of medicinal materials in Northeast Asian traditional medicine. BMC Complement Med Ther. 2024 Jan 16;24(1):40. 10.1186/s12906-023-04331-y 38229051 PMC10790428

[213] Naghizadeh A, Hamzeheian D, Akbari S, Mohammadi F, Otoufat T, Asgari S, et al. UNaProd: a universal natural product database for materia medica of Iranian traditional medicine. Evid Based Complement Alternat Med. 2020 May 13;2020:3690781. 10.1155/2020/3690781 32454857 PMC7243028

[214] Clausen J, Albrecht H, Mathie RT. Veterinary clinical research database for homeopathy: placebo-controlled trials. Complement Ther Med. 2013 Apr;21(2):115–20. 10.1016/j.ctim.2012.11.009 23497815

[215] Nguyen-Vo TH, Le T, Pham D, Nguyen T, Le P, Nguyen A, et al. VIETHERB: a database for Vietnamese herbal species. J Chem Inf Model. 2019 Jan 28;59(1):1–9. 10.1021/acs.jcim.8b00399 30407009

[216] Fan KW. Online research databases and journals of Chinese medicine. J Altern Complement Med. 2004 Dec;10(6):1123–8. 10.1089/acm.2004.10.1123 15674011

[217] Song Y, Yang Y, Xu L, Bian C, Xing Y, Xue H, et al. The burdock database: a multi-omic database for Arctium lappa, a food and medicinal plant. BMC Plant Biol. 2023 Feb 9;23(1):86. 10.1186/s12870-023-04092-3 36759759 PMC9909940

[218] Corson TW, Crews CM. Molecular understanding and modern application of traditional medicines: triumphs and trials. Cell. 2007 Sep 7;130(5):769–74. 10.1016/j.cell.2007.08.021 17803898 PMC2507744

[219] Patwardhan B, Wieland LS, Aginam O, Chuthaputti A, Ghelman R, Ghods R, et al. Evidence-based traditional medicine for transforming global health and well-being. J Ayurveda Integr Med. 2023 Jul–Aug;14(4):100790. 10.1016/j.jaim.2023.100790 37562183 PMC10432791

[220] Firenzuoli F, Gori L. Herbal medicine today: clinical and research issues. Evid Based Complement Alternat Med. 2007 Sep;4(Suppl 1):37–40. 10.1093/ecam/nem096 18227931 PMC2206236

[221] Greenwood M, Lindsay NM. A commentary on land, health, and Indigenous knowledge(s). Glob Health Promot. 2019 Apr;26(3 Suppl):82–6. 10.1177/1757975919831262 30964411

[222] Abuduxike G, Aljunid SM. Development of health biotechnology in developing countries: can private-sector players be the prime movers? Biotechnol Adv. 2012 Nov-Dec;30(6):1589–601. 10.1016/j.biotechadv.2012.05.002 22617902

[223] WHO traditional medicine global summit 2023 meeting report: Gujarat Declaration [internet]. Gujarat: World Health Organization; 2023. Available from: https://cdn.who.int/media/docs/default-source/who-global-traditional-medicine-centre/1st-tmg-summit/first-who-tm-global-summit-report.pdf [cited 2025 Feb 3].

